# Dysregulation of endometrial stromal serotonin homeostasis leading to abnormal phosphatidylcholine metabolism impairs decidualization in patients with recurrent implantation failure

**DOI:** 10.1093/hropen/hoae042

**Published:** 2024-06-20

**Authors:** Jiao Tian, Zhe Zhang, Jie Mei, Na Kong, Yuan Yan, Xiaoyue Shen, Jidong Zhou, Yang Zhang, Nannan Kang, Xin Zhen, Lijun Ding, Guijun Yan, Haixiang Sun, Xiaoqiang Sheng

**Affiliations:** Center for Reproductive Medicine and Obstetrics and Gynecology, Affiliated Drum Tower Hospital, Medical School of Nanjing University, Nanjing, China; State Key Laboratory of Pharmaceutical Biotechnology, Nanjing University, Nanjing, China; Center for Reproductive Medicine and Obstetrics and Gynecology, Affiliated Drum Tower Hospital, Medical School of Nanjing University, Nanjing, China; Center for Molecular Reproductive Medicine, Nanjing University, Nanjing, China; Center for Reproductive Medicine and Obstetrics and Gynecology, Affiliated Drum Tower Hospital, Medical School of Nanjing University, Nanjing, China; Center for Reproductive Medicine and Obstetrics and Gynecology, Affiliated Drum Tower Hospital, Medical School of Nanjing University, Nanjing, China; Center for Reproductive Medicine and Obstetrics and Gynecology, Affiliated Drum Tower Hospital, Medical School of Nanjing University, Nanjing, China; Center for Reproductive Medicine and Obstetrics and Gynecology, Affiliated Drum Tower Hospital, Medical School of Nanjing University, Nanjing, China; Center for Reproductive Medicine and Obstetrics and Gynecology, Affiliated Drum Tower Hospital, Medical School of Nanjing University, Nanjing, China; Center for Molecular Reproductive Medicine, Nanjing University, Nanjing, China; Center for Reproductive Medicine and Obstetrics and Gynecology, Affiliated Drum Tower Hospital, Medical School of Nanjing University, Nanjing, China; Center for Molecular Reproductive Medicine, Nanjing University, Nanjing, China; Center for Reproductive Medicine and Obstetrics and Gynecology, Affiliated Drum Tower Hospital, Medical School of Nanjing University, Nanjing, China; Center for Molecular Reproductive Medicine, Nanjing University, Nanjing, China; Center for Reproductive Medicine and Obstetrics and Gynecology, Affiliated Drum Tower Hospital, Medical School of Nanjing University, Nanjing, China; Center for Molecular Reproductive Medicine, Nanjing University, Nanjing, China; Center for Reproductive Medicine and Obstetrics and Gynecology, Affiliated Drum Tower Hospital, Medical School of Nanjing University, Nanjing, China; Center for Molecular Reproductive Medicine, Nanjing University, Nanjing, China; Center for Reproductive Medicine and Obstetrics and Gynecology, Affiliated Drum Tower Hospital, Medical School of Nanjing University, Nanjing, China; Center for Molecular Reproductive Medicine, Nanjing University, Nanjing, China; Center for Reproductive Medicine and Obstetrics and Gynecology, Affiliated Drum Tower Hospital, Medical School of Nanjing University, Nanjing, China; Center for Molecular Reproductive Medicine, Nanjing University, Nanjing, China; State Key Laboratory of Reproductive Medicine, Nanjing Medical University, Nanjing, China; Center for Reproductive Medicine and Obstetrics and Gynecology, Affiliated Drum Tower Hospital, Medical School of Nanjing University, Nanjing, China; Center for Reproductive Medicine, The Second Affiliated Hospital and Yuying Children’s Hospital of Wenzhou Medical University, Wenzhou, China

**Keywords:** serotonin, monoamine oxidase, endometrial decidualization, recurrent implantation failure, phosphatidylcholine

## Abstract

**STUDY QUESTION:**

Does abnormal serotonin homeostasis contribute to impaired endometrial decidualization in patients with recurrent implantation failure (RIF)?

**SUMMARY ANSWER:**

Abnormal serotonin homeostasis in patients with RIF, which is accompanied by decreased monoamine oxidase (MAO) expression, affects the decidualization of endometrial stromal cells and leads to embryo implantation failure.

**WHAT IS KNOWN ALREADY:**

Previous studies have indicated that the expression of MAO, which metabolizes serotonin, is reduced in the endometrium of patients with RIF, and serotonin can induce disruption of implantation in rats. However, whether abnormal serotonin homeostasis leads to impaired decidualization in patients with RIF and, if so, the mechanism involved, remains unclear.

**STUDY DESIGN, SIZE, DURATION:**

Endometrial samples from 25 patients with RIF and 25 fertile patients were used to investigate the expression levels of monoamine oxidase A (MAOA), monoamine oxidase B (MAOB), and serotonin. We isolated human endometrial stromal cells to investigate the role of MAOA, MAOB, and serotonin in inducing decidualization *in vitro* and further explored the underlying mechanism using RNA-sequencing (RNA-seq) and liquid chromatography-mass spectrometry (LC/MS) analyses.

**PARTICIPANTS/MATERIALS, SETTING, METHODS:**

The levels of serotonin in the endometrium of patients with RIF were detected by ELISA and immunohistofluorescence, and the key genes involved in abnormal serotonin metabolism were analyzed via combination with single-cell sequencing data. The effects of MAOA or MAOB on the decidualization of stromal cells were investigated using an *in vitro* human endometrial stromal cell-induced decidualization model and a mouse artificially induced decidualization model. The potential mechanisms by which MAOA and MAOB regulate decidualization were explored by RNA-seq and LC/MS analysis.

**MAIN RESULTS AND THE ROLE OF CHANCE:**

We found that women with RIF have abnormal serotonin metabolism in the endometrium and attenuated MAO in endometrial stromal cells. Endometrial decidualization was accompanied by increased MAO *in vivo* and *in vitro*. However attenuated MAO caused an increased local serotonin content in the endometrium, impairing stromal cell decidualization. RNA-seq and LC/MS analyses showed that abnormal lipid metabolism, especially phosphatidylcholine metabolism, was involved in the defective decidualization caused by MAO deficiency. Furthermore, decidualization defects were rescued by phosphatidylcholine supplementation.

**LARGE SCALE DATA:**

RNA-seq information and raw data can be found at NCBI Bioproject number PRJNA892255.

**LIMITATIONS, REASONS FOR CAUTION:**

This study revealed that impaired serotonin metabolic homeostasis and abnormally reduced MAO expression were among the reasons for RIF. However, the source and other potential functions of serotonin in the endometrium remain to be further explored.

**WIDER IMPLICATIONS OF THE FINDINGS:**

This study provides new insights into the mechanisms of serotonin homeostasis in human endometrial decidualization and new biomarkers or targets for the treatment of patients with RIF.

**STUDY FUNDING/COMPETING INTEREST(S):**

X. Sheng is supported by grants from the National Natural Science Foundation of China (82001629), the Wenzhou Basic Public Welfare Research Project (Y20240030), the Youth Program of Natural Science Foundation of Jiangsu Province (BK20200116), and Jiangsu Province Postdoctoral Research Funding (2021K277B). H.S. is supported by grants from the National Natural Science Foundation of China (82030040). G.Y. is supported by grants from the National Natural Science Foundation of China (82171653). The authors declare no conflicts of interest.

WHAT DOES THIS MEAN FOR PATIENTS? Recurrent implantation failure (RIF) is a defined clinical disorder in which patients who have good-quality embryos transferred fail to achieve pregnancy repeatedly. Many women experiencing infertility also suffer from anxiety and depression, which could potentially be linked to lower success rates of pregnancy following IVF-embryo transfer. Mental health disorders including anxiety, depression, and stress are associated with imbalances of monoamine neurotransmitters such as norepinephrine, serotonin, and dopamine. In the present study, we found that women with RIF had higher levels of serotonin and lower levels of monoamine oxidase (which breaks down serotonin) in the endometrium; these changes impair decidualization. Decidualization is a phase when the endometrial stromal fibroblasts transform into decidual cells to support embryo implantation and provide nutrients. We observed an increased expression of monoamine oxidase in the endometrium during decidualization. Further investigation revealed that the higher serotonin levels or lower monoamine oxidase levels hindered decidualization, which was accompanied by abnormal phospholipid metabolism. Additionally, we found that supplementation of phosphatidylcholine, a type of phospholipid, was able to rescue the decidualization defects. In summary, our study provides valuable insights into the mechanisms underlying the serotonin balance in endometrial decidualization and proposes potential treatment avenues for patients experiencing RIF.

## Introduction

Embryo implantation is a complex and critical process involving interactions between a developing embryo and the receptive endometrium. During the implantation window, embryos undergo apposition and adhere to the endometrial epithelium, while stromal cells differentiate into decidual cells, facilitating trophoblast invasion (Niringiyumukiza *et al.*, 2018). Although the advancements of ART have enabled many infertile patients to conceive, the failure rate of embryo implantation remains high at ∼50% even with good-quality embryos, highlighting the importance of uncovering the mechanisms underlying impaired endometrial receptivity (Hill *et al.*, 2020).

Recurrent implantation failure (RIF) is clinically defined as the inability to achieve pregnancy after at least four good-quality embryo transfers over a minimum of three fresh or frozen embryo transfer cycles. RIF affects ∼10% of infertile patients undergoing IVF and embryo transfer (IVF-ET) worldwide (Cimadomo *et al.*, 2021). RIF has a complex etiology and is a challenging clinical problem. Previous studies have shown that patients experiencing ART failure often exhibit increased anxiety and depression (Maroufizadeh *et al.*, 2015; Milazzo *et al.*, 2016). However, the relationship between failure of embryo implantation in RIF and psychological stress is still not clear and requires further clarification.

High levels of psychological stress have been shown to be associated with an increased risk of infertility (Coughlan *et al.*, 2014; Lynch *et al.*, 2014). Women with anxiety or depression have lower pregnancy rates and live birth rates after IVF-ET (Cesta *et al.*, 2016). However, the specific mechanism of how anxiety or depression affects pregnancy is not clear. Anxiety and depression are associated with abnormal levels of neurotransmitters such as norepinephrine, serotonin, and dopamine, and their receptors are widely distributed in the human endometrium (Gibson, 2018; Xu *et al.*, 2021). Several studies have shown that abnormal elevation of monoamine neurotransmitters such as epinephrine (also known as adrenalin) and serotonin can inhibit embryo implantation and decidualization in mice (Mitchell *et al.*, 1983; Wu *et al.*, 2019). However, there is still limited evidence regarding whether monoamine neurotransmitter homeostasis is a contributing factor to reproductive failure associated with mental health conditions and, if so, the specific mechanism is not clear.

Monoamine oxidase (MAO) is a central regulatory molecule in the metabolism of monoamine neurotransmitters, which can catalyze the oxidative deamination of monoamine neurotransmitters (Jones and Raghanti, 2021). Two isoenzymes, monoamine oxidase A (MAOA) and monoamine oxidase B (MAOB), which are encoded by different genes on the X chromosome, are highly conserved in most mammals (Derry *et al.*, 1989). Many mental health disorders are related to MAO dysfunction, and anxiety and depression involve metabolic imbalances of monoamine neurotransmitters including epinephrine, serotonin, and dopamine (Bortolato *et al.*, 2009; Thomas *et al.*, 2015). Our previous study revealed that decreased MAOA expression in endometrial epithelial cells leads to elevated serotonin, excessive cell proliferation, and decreased endometrial receptivity (Tian *et al.*, 2021). However, the function of MAO and serotonin homeostasis in endometrial stromal cells remains unclear.

In the present study, we aimed to investigate the effects and mechanisms of serotonin and MAO in patients with RIF. We demonstrated that the serotonin metabolic pathway was impaired due to attenuated MAO in the endometrium of patients with RIF. A decrease in MAO leads to an imbalance in serotonin homeostasis, resulting in impaired decidualization of either human endometrial stromal cells (hESCs) or mouse endometrium. Moreover, RNA-sequencing (RNA-seq) and liquid chromatography-mass spectrometry (LC/MS) analyses revealed that MAO participates in the decidualization of hESCs by regulating phospholipid metabolism, particularly phosphatidylcholine (PC). PC could rescue serotonin-suppressed endometrial stromal cell decidualization, accompanied by the upregulation of forkhead box O1 (FOXO1). Additionally, overexpression of FOXO1 also rescues impaired decidualization caused by attenuated MAO or excess serotonin and improves embryo implantation. Thus, this study revealed the role of serotonin homeostasis and appropriate MAO levels in regulating uterine decidualization, providing a new perspective on infertility linked to anxiety and depression.

## Materials and methods

### Patient and sample collection

The patients involved in this study were recruited from the Reproductive Center of the Affiliated Drum Tower Hospital of Nanjing University Medical School from September 2019 to October 2023. A total of 25 patients with RIF and 25 control patients were enrolled. Endometrium from patients with RIF and control patients was collected in the mid-secretory phase (5–7 days after ovulation, as monitored by ultrasound). RIF was defined as a failure to achieve pregnancy after at least four good-quality embryo transfers over a minimum of three fresh or frozen embryo transfer cycles. Patients with adenomyosis, endometriosis, intrauterine adhesions, uterine malformations, chromosomal abnormalities, or recurrent miscarriage were excluded. The endometrium in the proliferative phase was collected within 7 days after the end of menstruation, and the endometrium in the secretory phase was collected within 5–7 days after ovulation monitored by ultrasound. Both groups of women had normal menstrual cycles. This study was approved by the Research and Ethics Committee of Drum Tower Hospital (2013-081-01), and all patients signed written informed consent before surgery. The clinical characteristics of women enrolled in the present study are shown in Table 1.

**Table 1.** The clinical characteristics of patients with recurrent implantation failure and control patients enrolled in the present study.

	Control patients (n = 25)	RIF patients (n = 25)	*P* value
Age (years)	29.52 ± 3.19	28.84 ± 3.64	0.4855
BMI (kg/m^2^)	21.35 ± 2.64	22.89 ± 3.78	0.1009
FSH (mIU/ml)	8.25 ± 1.86	7.84 ± 2.25	0.4907
LH (mIU/ml)	5.91 ± 2.89	4.46 ± 2.82	0.0797
Oestradiol (pg/ml)	36.33 ± 15.56	40.92 ± 18.19	0.3419
AFC	15.96 ± 4.56	16.08 ± 4.99	0.9297
Number of embryos transferred	1.80 ± 0.58	5.36 ± 1.55	<0.0001

RIF, recurrent implantation failure; AFC, antral follicle count. Results are expressed as mean±SD.

### Single-cell RNA and Visium data analysis

Single-cell RNA (scRNA) data from primary endometrial stromal cells along a decidual time-course (Day 0, Day 2, Day 4, Day 6, Day 8, WD) *in vitro* were downloaded from GSE127918 (Lucas *et al.*, 2020). Endometrial scRNA data from patients with RIF and healthy controls were downloaded from GSE183837 (Lai *et al.*, 2022). scRNA data from endometrium at different phases of the menstrual cycle were downloaded from GSE111976 (Wang *et al.*, 2020). The scRNA data were analyzed with Seurat V4.1.1 (Stuart *et al.*, 2019). The Seurat object was created based on two filtering parameters of ‘min.cells = 5’ and ‘low.thresholds = 200’, and the exorbitant number of unique genes detected in each cell (i.e. ‘nFeature_RNA’) was adjusted in each sample to eliminate the empty drops and dying cells and potential doublets/multiplets from subsequent analyses. Then, the multiple samples were processed with ‘harmony’ (Korsunsky *et al.*, 2019). Following the normalization and scaling steps, using the uniform manifold approximation and projection (UMAP, a visualized method for cell clustering of high-dimensional transcriptomic data) technique (Becht *et al.*, 2018), a series of commands were executed to visualize cell clusters, including the ‘RunUMAP’ function with a proper combination of the ‘resolution’ and ‘dims.use’, ‘FindNeighbors’ and ‘FindClusters’ functions to conduct cell clustering. To identify canonical cell cluster marker genes, the ‘FindAllMarkers’ function was used to identify conserved marker genes in clusters with default parameters (Hao *et al.*, 2021). We identified eleven clusters that could be grouped into seven main cellular categories based on their expression of known markers (Garcia-Alonso *et al.*, 2021): (i) decidualized endometrial (dS) cells; (ii) non-decidualized endometrial (eS) cells; (iii) immune cells; (iv) fibroblasts expressing C7 (fibroblasts C7); (v) epithelial cells; (vi) perivascular (PV) cells; and (vii) endothelial cells. Serotonin-related metabolic pathway analysis was performed using the scMetabolism (Wu *et al.*, 2022) package with minor modification and reconstructed network modules from scFEA (Alghamdi *et al.*, 2021). The R code of serotonin-related metabolic pathway analysis can be found in https://git.nju.edu.cn/littleflea/serotonin-related-metabolic-pathway-analysis. The 10x Visium data from full-thickness uteri *in vivo* were downloaded from E-MTAB-9260 (Garcia-Alonso *et al.*, 2021), the unique molecular identifier count matrix processed using the R package Seurat V4.1.1 (Stuart *et al.*, 2019) and the data were normalized by SCTransform (Hafemeister and Satija, 2019) to account for variance in sequencing depth across data points. ‘SpatialFeaturePlot’ function was used to display the spatial expression and distribution of different genes.

### Cell culture and transfection

To investigate the role of MAO in human endometrial decidualization, we used an *in vitro* decidualization model of hESCs. Human endometrial stromal cells were isolated from endometrium as previously described (Sun *et al.*, 2009). Briefly, the endometrium was minced with scissors after rinsing with phosphate-buffered saline (PBS) (Corning, Corning, NY, USA). Then, the minced endometrium was digested with 0.15% collagenase I (Worthington Biochemical Corp, Lakewood, CO, USA) for 30 min with gentle pipetting every 10 min. After digestion was stopped with serum-containing medium, the digested tissues were centrifuged at 100*g* for 5 min, and the supernatant was aspirated and discarded. The precipitate was resuspended and filtered through a 40-μM cell strainer (Corning). The filtered cells were cultured in DMEM/F12 (Gibco, Grand Island, NY, USA) medium containing 10% fetal bovine serum (Gibco) at 37°C in 5% CO_2_. MAOA or MAOB siRNA (Ruibo, Guangzhou, China) was transfected into hESCs utilizing Lipofectamine 3000 (Invitrogen, Carlsbad, CA, USA). The decidualization of hESCs was induced with 0.5 mM 8-bromoadenosine 3′,5′-cyclic monophosphate (8Br-cAMP) (Sigma-Aldrich, St Louis, MO, USA) and 1 μM medroxyprogesterone acetate (MPA) (Sigma Aldrich) in phenol red-free DMEM/F12 medium (Gibco BRL/Invitrogen, Carlsbad, CA, USA) containing 2.5% charcoal/dextran-treated serum (Thermo Fisher Scientific, Waltham, MA, USA).

### RNA isolation and quantitative real-time PCR

Total RNA was extracted from cells or endometrial tissue using TRIzol reagent (Life Technologies, Carlsbad, CA, USA). Samples of 1 µg of total RNA were reverse transcribed into cDNA using the PrimeScript RT reagent kit (Abm, Richmond, BC, Canada) according to the manufacturer’s instructions. Quantitative real-time PCR was conducted on a qTOWER³ real-time PCR thermal cycler (Analytik, Jena, Germany) with a SYBR Green PCR kit (Vazyme, Nanjing, China). The primer sequences listed in Supplementary Table S1 were used to amplify the indicated genes.

### Western immunoblotting analysis

Patient endometrium was lysed with radioimmunoprecipitation assay (RIPA) buffer (50 mM Tris-HCl, pH 7.6; 150 mM NaCl; and 1.0% NP-40) containing protease inhibitor cocktail (Roche, Basel, Switzerland) for 30 min at 4°C (Sheng *et al.*, 2022). The protein concentration was measured with a bicinchoninic acid protein assay reagent (Thermo Fisher Scientific). Proteins were electrophoresed through 10% sodium dodecyl sulfate-polyacrylamide gel electrophoresis gels and then transferred to 0.22 μm polyvinylidene fluoride (PVDF) membranes (Millipore, Billerica, MA, USA). The PVDF membranes were blocked with 5% skimmed milk (Bio-Rad, Hercules, CA, USA) and then incubated with primary antibodies against MAOA (1:2000, ab126751, Abcam, Cambridge, UK), MAOB (1:2000, NBP1-32426, Novus Biologicals, Centennial, CO, USA), FOXO1 (1:2000, 2880S, CST, Danvers, MA, USA), phosphatidylethanolamine *N*-methyltransferase (PEMT) (1:2000, PA5-98046, Invitrogen), and GAPDH (1:10 000, AP0063, Bioworld, Irving, TX, USA). Detection was conducted using a chemiluminescence horseradish peroxidase substrate kit (Millipore).

### ELISA analysis

ELISA analysis was used to detect endometrial serotonin levels of women with RIF. Patient endometrium (∼0.1 g) was lysed using 200 μl RIPA buffer (50 mM Tris-HCl, pH 7.6; 150 mM NaCl; and 1.0% NP-40). After the protein concentration was subsequently measured, these samples were diluted to a concentration of 1 μg total protein/μl by adding RIPA buffer and the serotonin levels were then detected using an ELISA kit (Elabscience, Wuhan, China) according to the manufacturer’s instructions.

### Cellular immunofluorescence

Cellular immunofluorescence was used to evaluate the morphological changes of hESCs during decidualization *in vitro*. Human endometrial stromal cells were grown in 24-well plates and fixed in 4% paraformaldehyde for 30 min at room temperature. The cells were permeabilized with 0.5% Triton X-100 for 10 min after rinsing with PBS. Non-specific binding sites were blocked with 3% bovine serum albumin (BSA) at 37°C for 30 min. The cells were incubated with antibodies against MAOA (1:200, ab126751, Abcam) and MAOB (1:200, NBP1-32426, Novus Biologicals) overnight at 4°C. After the cells were washed with PBST, they were incubated with donkey anti-rabbit secondary antibody (1:500, A21207, Invitrogen) for 1 h at room temperature. The cytoskeleton was stained with phalloidin (1:200, P5282, Sigma-Aldrich) for 1 h at room temperature, and the cell nuclei were stained with DAPI (G1012, Servicebio, Wuhan, China) for 10 min. Finally, images were captured with a fluorescence microscope (Leica, Wetzlar, Germany).

### Immunohistochemistry

Endometrium and mouse uterine tissue were fixed overnight in 10% formalin solution, dehydrated with ascending ethanol solutions, and then embedded in paraffin. The tissue samples were serially sectioned to a thickness of 3 μm, dewaxed with xylene, and rehydrated through a graded alcohol series. Endogenous peroxidase activity was blocked with 3% hydrogen peroxide for 10 min. Then, antigen retrieval was performed through treatment with high pressure at 120°C for 15 min in sodium citrate buffer (pH 6.0). The sections were blocked with goat serum, incubated with antibodies against MAOA (1:200, ab126751, Abcam), MAOB (1:200, NBP1-32426, Novus Biologicals), FOXO1 (1:300, 2880s, CST), and heart and neural crest derivatives expressed 2 (HAND2) (1:1000, 200040, Abcam) at 4°C overnight, then incubated with goat anti-rabbit secondary antibody, and finally stained with 3,3′-diaminobenzidine (DAB) (Zsbio, Beijing, China) and counterstained with hematoxylin. Non-specific rabbit IgG was used as a negative control, and the other procedures were the same as those for the experimental group.

### Construction of adenovirus

Adenovirus was constructed to knock down the expression of MAOA or MAOB in the mouse uterus. The shRNA adenovirus vector was constructed by inserting the sense sequence of the interference target sequence (*Maoa*: GCTCCAATTTCAATCACTC, *Maob*: GTGGAACCATGGTTATCGA), hairpin loop, antisense sequence, and terminator between the *Bam*HI and *Eco*RI sites of the pacAd5-U6-GFP shuttle plasmid according to the manufacturer’s recommendation of RAPAd^®^ shRNA Adenoviral Expression Systems (Cell Biolabs Inc, San Diego, CA, USA). Adenoviruses harboring the full-length *FOXO1* gene (Ad-Flag-FOXO1, NCBI Reference Sequence: NM_002015.4 (human) and NM_019739.3 (mouse)) were generated using an AdMax (Microbix, Mississauga, ON, Canada) system according to the manufacturer’s recommendations. The virus was packaged and amplified in HEK293A cells and purified using CsCl banding.

### Animals

All animal experiments were conducted under the guidance of the Laboratory Animal Management Committee (Jiangsu Province, China), and approved by the Institutional Animal Care and Use Committee of Nanjing Drum Tower Hospital (SCXK 2019-0059). ICR mice were raised in the Experimental Animal Center of Nanjing Drum Tower Hospital (Nanjing, China) under specific pathogen-free conditions in a controlled environment of 20 ± 2°C, a 12:12 h light: dark cycle, and 50–70% humidity, with food and water provided *ad libitum*. Artificially induced mouse endometrial decidualization was used to investigate the effects of knocking down the expression levels of MAOA or MAOB on decidualization. Six-week-old female ICR mice were ovariectomized, and the uterine horns were injected bilaterally with 20 μl of adenovirus, followed by a 2-week rest period. Two weeks later, the mice were injected with adenovirus via the tail vein and rested for two days. Afterwards, 50 µl of sesame oil (Sigma, St Louis, MO, USA) containing 100 ng oestradiol (Sigma-Aldrich) was subcutaneously injected for three consecutive days, followed by two days of rest. Then, 50 µl of sesame oil containing 1 mg of progesterone (Sigma-Aldrich) and 10 ng of oestradiol was subcutaneously injected for three consecutive days. Six hours after the third combined administration of estrogen and progesterone, 20 μl of sesame oil was injected into one of the uterine horns (unilaterally) to simulate the decidualization of the endometrium. Subcutaneous injections of estrogen and progesterone were continued for 4 days. Mice were sacrificed 6 h after the last hormone injection. To explore whether overexpression of FOXO1 could rescue embryo implantation failure caused by decreased MAOA levels, we conducted the following experiments. Mice were injected with 20 μl of adenovirus (20 μl control adenovirus/10 μl control adenovirus + 10 μl shMAOA adenovirus/10 μl shMAOA adenovirus+10 μl Ad-FOXO1 adenovirus) in the uterine horns bilaterally at 1.5 days post-coitum (dpc1.5), and Chicago blue dye was injected into the tail vein at dpc4.5 for observation of the embryo implantation sites.

### RNA-seq and data analysis

RNA-seq was performed to explore the mechanism of impaired decidualization after MAO knockdown in hESCs. Total RNA of hESCs was extracted with TRIzol reagent and 1 μg RNA was used for library construction with a VAHTS Universal V6 RNA-seq Library Prep Kit for Illumina (NR604-01, Vazyme, Nanjing, China). Library sequencing and analysis were performed by Novogene (Beijing, China) and iDEP.93(2016). Gene ontology (GO) enrichment analysis was performed with Metascape (Zhou *et al.*, 2019). Heatmaps and Venn diagrams were generated using TBtools (Chen *et al.*, 2020).

### Spectrometry analysis of supernatant

Supernatants of hESCs during decidualization were collected for metabolite analysis. The untargeted metabolomics LC/MS analysis was conducted by OE Biotech Co., Ltd (Shanghai, China). The analytic instrument used in this experiment was an LC/MS system composed of a Nexera UPLC ultrahigh-performance liquid phase tandem QE high-resolution mass spectrometer.

## Results

### Increased endometrial serotonin expression in patients with RIF

**Figure 1.**
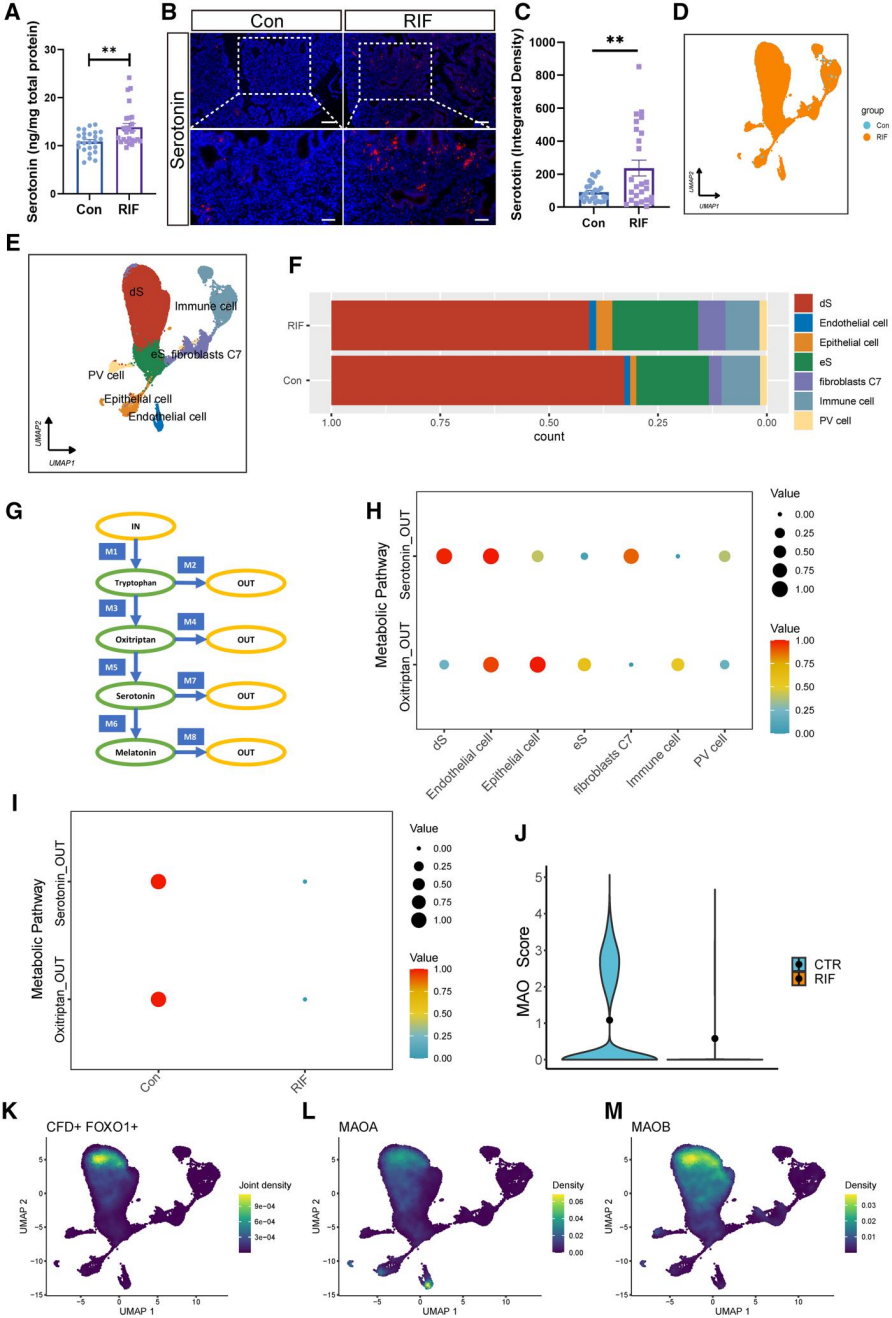
**Impaired serotonin metabolism in the endometrium patients with recurrent implantation failure**. (**A**) Serotonin expression in endometrium from patients with RIF and control patients was measured by ELISA (n = 25, **P<0.01, statistically significant by Student’s *t*-test). (**B** and **C**) Immunofluorescence analysis showing the endometrial serotonin expression levels of RIF and control patients. Semiquantitative analysis of immunofluorescence was performed using ImageJ (n=25, scale bars=50 μm (bottom) and 100 μm (top), **P<0.01, statistically significant by Student’s *t*-test). (**D**) UMAP plot of clustering of endometrial cells color-coded according to groups (Control: blue; RIF: orange). (**E**) UMAP plot of clustering of endometrial cells, where each color represents a cluster of cells: decidualized endometrial stromal cells, endothelial cells, epithelial cells, non-decidualized endometrial stromal cells, fibroblasts, immune cells, and perivascular cells. (**F**) Proportions of dS cells, endothelial cells, epithelial cells, eS cells, fibroblasts, immune cells, and PV cells in the endometrium of patients with RIF and healthy controls. (**G**) Metabolic network of biosynthesis and metabolism of the neurotransmitter serotonin in human. This network can be subdivided into eight modules (M1: IN_Tryptophan, M2: Tryptophan_OUT, M3: Tryptophan_Oxitriptan, M4: Oxitriptan_OUT, M5: Oxitriptan_Serotonin, M6: Serotonin_Melatonin, M7: Serotonin_OUT, M8: Melatonin_OUT). (**H**) Dot plots of specific analysis of Oxitriptan_OUT and Serotonin_OUT modules in serotonin metabolism in different cell types of the endometrium from patients with RIF or healthy controls. (**I**) Dot plots of specific analysis of Oxitriptan_OUT and Serotonin_OUT modules in serotonin metabolism enriched in dS cells. (**J**) The expression levels of MAO were scored in dS cells in the endometrium of patients with RIF or healthy controls. (**K**) UMAP visualization showed marker genes (CFD and FOXO1) of dS cells. (**L** and **M**) UMAP visualization showed the expression of MAOA and MAOB in dS cells. RIF, recurrent implantation failure; UMAP, uniform manifold approximation and projection; scRNA-seq, single-cell RNA sequencing; dS cells, decidualized endometrial stromal cells; eS cells, non-decidualized endometrial stromal cells; PV cells, perivascular cells; MAO, monoamine oxidase; CFD, complement factor D; FOXO1, forkhead box O1; MAOA, monoamine oxidase A; MAOB, monoamine oxidase B.

To explore whether serotonin signaling is associated with early pregnancy failure, we examined the endometrial serotonin levels of women with RIF. ELISA and immunofluorescence revealed that serotonin concentrations were elevated in the endometrium of women with RIF (n = 25) (Fig. 1A–C). To explore the cause of the increased endometrial serotonin levels in patients with RIF, we further analyzed single-cell sequencing data from the endometrium during the implantation window in patients with RIF. Endometrial cells from patients with RIF and healthy controls were categorized as decidualized stromal cell (dS), non-decidualized stromal cell (eS), epithelial cell, immune cell, fibroblasts expressing C7 (fibroblasts C7), endothelial cell, or perivascular cell (PV cell) (Fig. 1D and E). The proportion of decidualized stromal cells was decreased in patients with RIF compared with that in healthy controls (Fig. 1F). Subsequently, we further analyzed changes in the serotonin metabolic pathway, which was divided into eight modules for the synthesis and metabolism of tryptophan, oxitriptan, serotonin, and melatonin, in patients with RIF (Fig. 1G). Two modules, oxitriptan and serotonin catabolism, were enriched in endometrial cells, while the other six modules were not (Fig. 1H and I). The catabolism of serotonin was significantly greater in the dSs than in the eSs (Fig. 1H), but was lower in the dSs of patients with RIF (Fig. 1I). Therefore, we analyzed the expression levels of serotonin catabolism-related MAOs, which were significantly decreased in the dSs of patients with RIF (Fig. 1J). FOXO1 and CFD (complement factor D) were used as molecular markers to identify the dSs (Fig. 1K), and MAOA and MAOB were highly expressed in the CFD^+^FOXO1^+^ cells (Fig. 1L and M). These results suggest that the abnormal serotonin levels in patients with RIF may be caused by the decreased expression of MAO in endometrial stromal cells.

### Decreased endometrial MAOA and MAOB expression in patients with RIF

**Figure 2.**
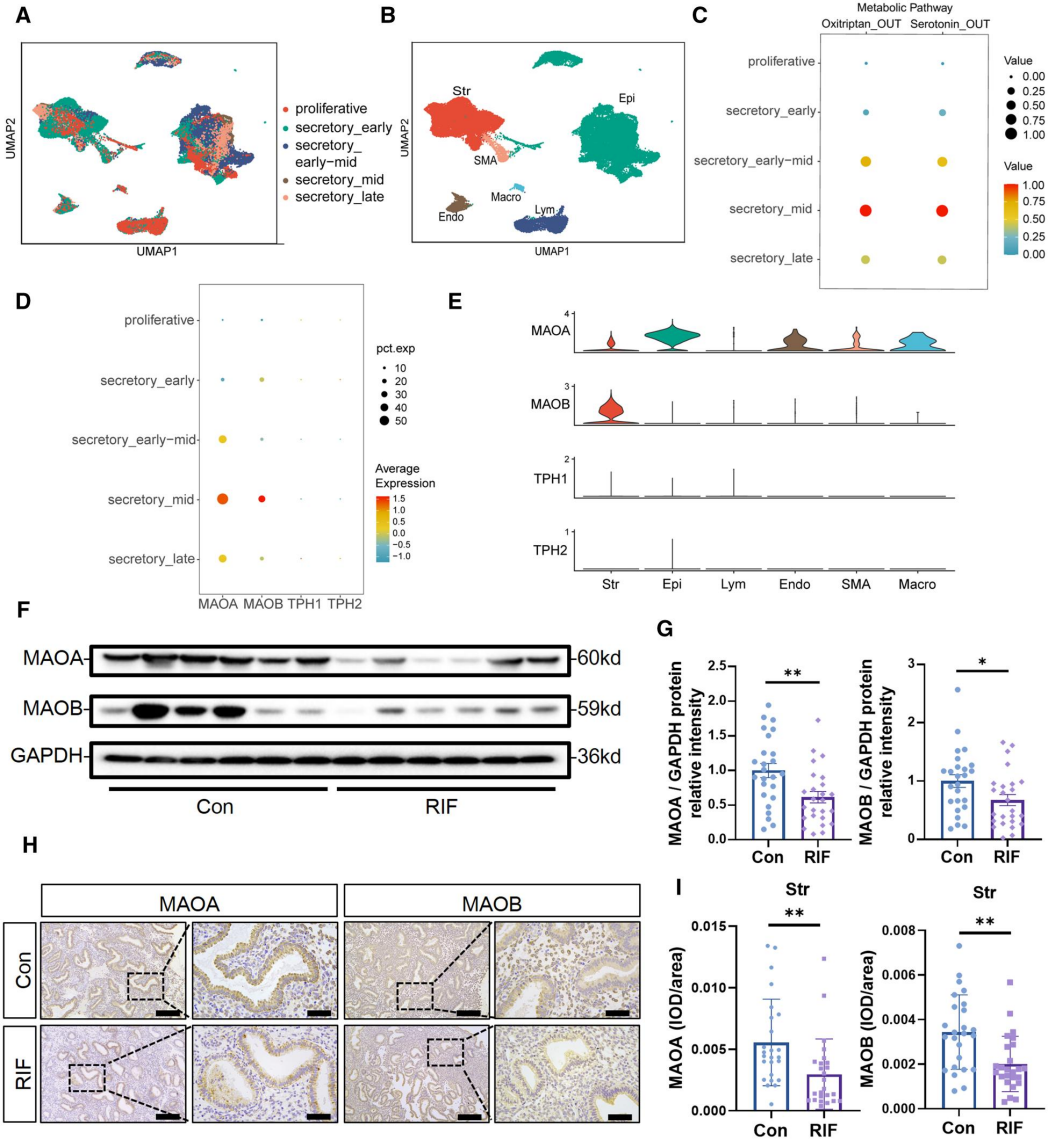
**Decreased endometrial monoamine oxidase expression in patients with recurrent implantation failure**. (**A**) UMAP plot of endometrial color-coded by different periods of the menstrual cycle, including proliferative phase, secretory_early phase, secretory_early-mid phase, secretory_mid phase, and secretory_late phase. (**B**) UMAP projections of clustering of endometrial cells color-coded by cell types, including stromal cells, epithelial cells, lymphocytes, endothelial cells, smooth muscle cells, and macrophages. (**C**) Dot plots of specific analysis of Oxitriptan_OUT and Serotonin_OUT modules in serotonin metabolism enriched in different periods of the menstrual cycle. (**D**) Dot plots of expression levels of serotonin degradation-related proteins (MAOA and MAOB) and serotonin synthesis-related proteins (TPH1 and TPH2) in the endometrium during different periods of the menstrual cycle. (**E**) Vlnplots of expression levels of MAOA, MAOB, TPH1, and TPH2 in different types of cells in mid-secretory phase. (**F**) Protein expression of MAOA and MAOB in RIF and control endometrium during mid-secretory phase. (**G**) Total MAOA and MAOB protein levels were normalized to total GAPDH levels in the western-blot analysis (n=25, *P<0.05, **P<0.01, statistically significant by Student’s *t*-test). (**H** and **I**) Immunohistochemistry illustrates MAOA and MAOB expression levels and localization in RIF and control endometrium during mid-secretory phase. Semiquantitative analysis of MAOA and MAOB expression levels in the endometrial stroma was performed using Image-Pro Plus (n=25, scale bars=200 μm (left) and 50 μm (right), **P<0.01, statistically significant by Student’s *t*-test). RIF, recurrent implantation failure; UMAP, uniform manifold approximation and projection; scRNA-seq, single-cell RNA sequencing; Str, stromal cells; Epi, epithelial cells; Lym, lymphocytes; Endo, endothelial cells; SMA, smooth muscle cells; Macro, macrophages; MAOA, monoamine oxidase A; MAOB, monoamine oxidase B; TPH1, tryptophan hydroxylase 1; TPH2, tryptophan hydroxylase 2.

To further clarify the cause of abnormal endometrial serotonin levels in RIF patients, we analyzed the single-cell sequencing data of endometrium at different menstrual periods to investigate changes in serotonin metabolism during the menstrual cycle (Fig. 2A and B). Two modules, oxitriptan and serotonin catabolism, were found to be similarly enriched in single-cell sequencing data of the menstrual cycle endometrium and were significantly elevated in the mid-secretory phase (Fig. 2C). Through analysis of key serotonin metabolism genes, we found that the serotonin synthesis-related proteins tryptophan hydroxylase 1 (TPH1) and tryptophan hydroxylase 2 (TPH2) were barely expressed during the proliferative and secretory phases, while the expression levels of MAOA and MAOB were significantly increased during the mid-secretory phase (Fig. 2D). In the mid-secretory phase, MAOA was expressed in stromal cells, epithelial cells, endothelial cells, and other cells, while MAOB was expressed in stromal cells (Fig. 2E). Since the serotonin synthetic enzyme TPH is barely expressed in the endometrium, we investigated the expression levels of serotonin metabolism-related MAO in the endometrium of patients with RIF. Western blotting showed that MAOA and MAOB expression levels were 0.5-fold lower in RIF endometrium than in control endometrium (n = 25) during the mid-secretory phase (Fig. 2F and G). As shown in Fig. 2H and I, the expression of MAOA and MAOB was significantly decreased in endometrial stromal cells of patients with RIF as determined by immunohistochemical staining (n = 25).

### Diagnostic significance of endometrial serotonin, MAOA, and MAOB levels

**Figure 3.**
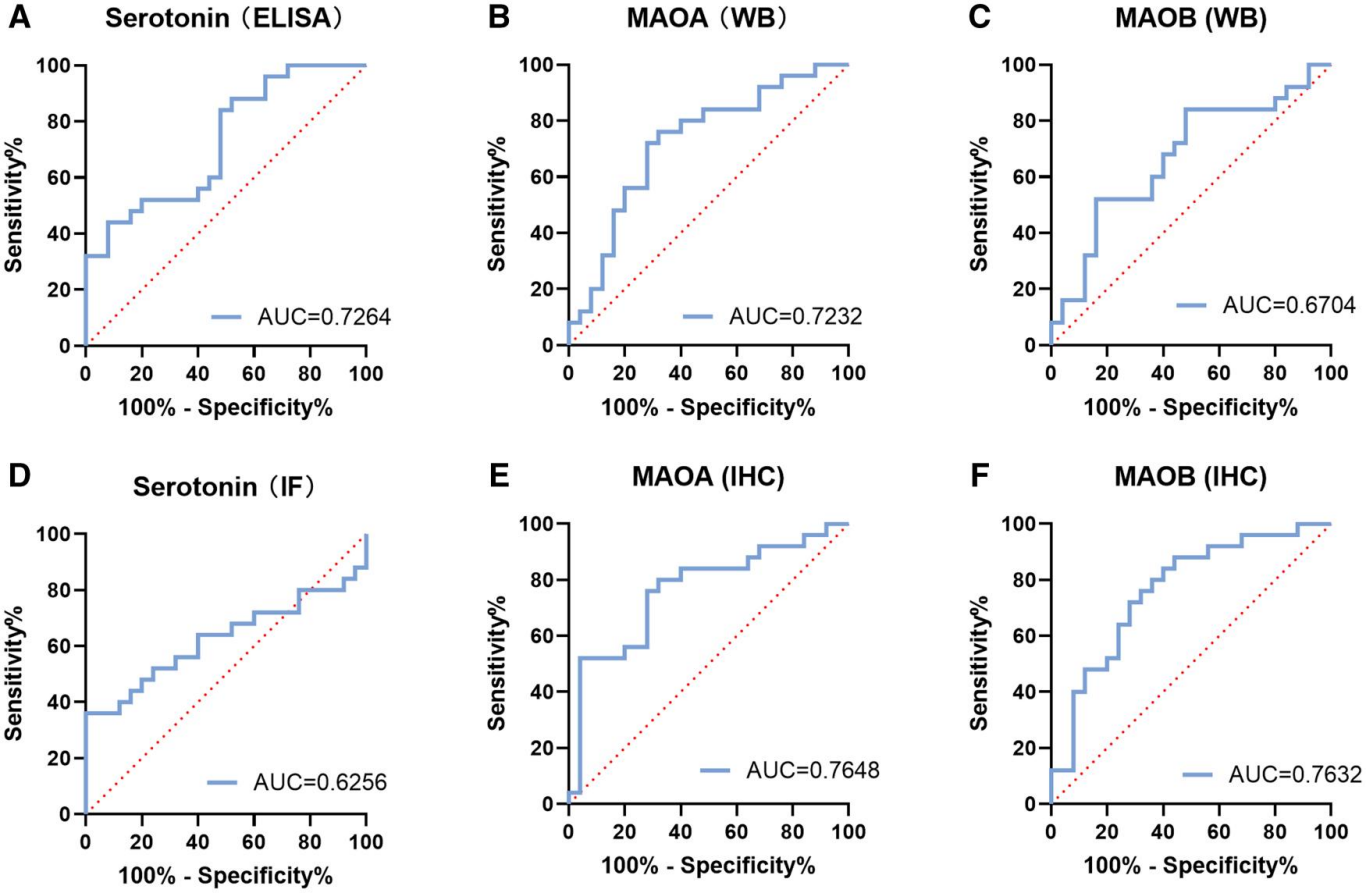
**Receiver operating characteristic curves estimating the diagnostic performance of endometrial serotonin, monoamine oxidase A, and monoamine oxidase B levels**. (**A**) ROC curves assessing the performance of endometrial serotonin levels detected by ELISA. (**B** and **C**) ROC curves assessing the performance of endometrial MAOA and MAOB levels detected by WB. (**D**) ROC curves assessing the performance of endometrial serotonin levels detected by IF. (**E** and **F**) ROC curves assessing the performance of endometrial stromal MAOA and MAOB levels detected by IHC. ROC curves, receiver operating characteristic curves; MAOA, monoamine oxidase A; MAOB, monoamine oxidase B; WB, western blot; IF, immunofluorescence; IHC, immunohistochemistry.

To determine whether endometrial serotonin, MAOA, and MAOB levels can be used as diagnostic markers for patients with RIF, receiver operating characteristic (ROC) analysis was performed to explore their diagnostic value for RIF (n = 25). The results showed that the expression levels of serotonin (detected by ELISA (Fig. 3A)), MAOA (detected by immunohistochemistry and western blot (Fig. 3B and E)) and MAOB (detected by immunohistochemistry (Fig. 3F)) have potential predictive value for RIF (area under curve (AUC) > 0.7). However, serotonin (detected by immunofluorescence (Fig. 3D)) and MAOB (detected by western blot (Fig. 3C)) had poor predictive values (AUC < 0.7). This result suggested that serotonin and MAO may play an important role in embryo implantation.

### MAO expression is elevated during decidualization

**Figure 4.**
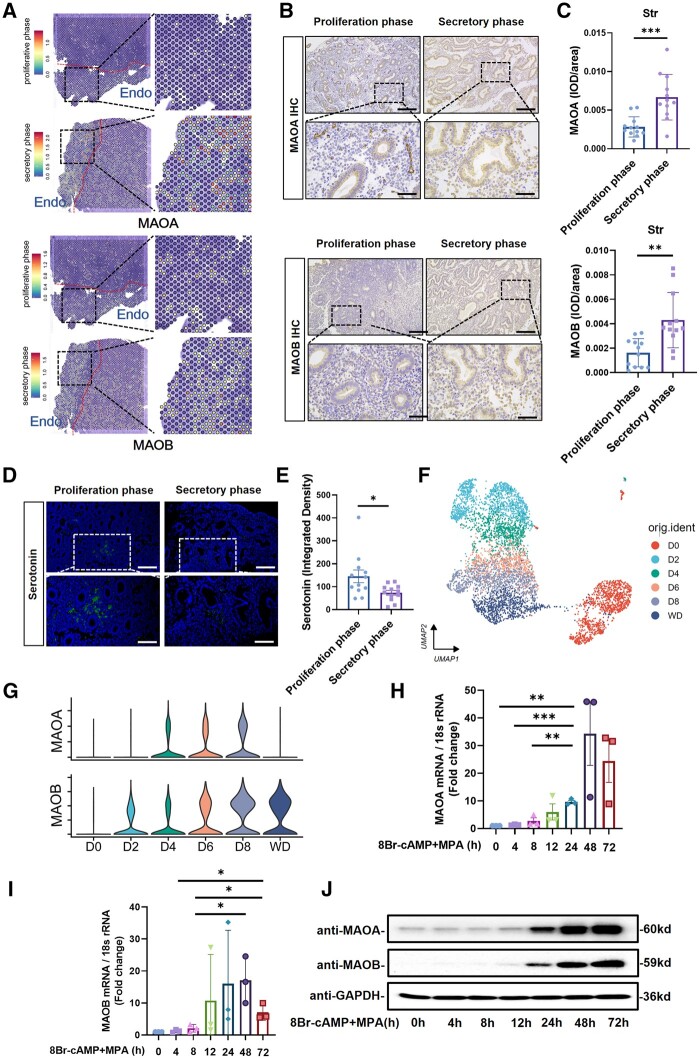
**Monoamine oxidase expression is elevated during endometrial decidualization**. (**A**) Visualization of the expression levels of MAOA and MAOB in the endometrium in the proliferation and secretory phases using spatial transcriptomics. (**B** and **C**) Immunohistochemistry shows MAOA and MAOB expression levels and localization in the endometrium in the proliferation and secretory phases. Semiquantitative analysis of the immunohistochemistry results was performed using Image-Pro Plus (n=12, scale bars=50 μm (bottom) and 200 μm (top)). (**D** and **E**) Immunofluorescence analysis showing the endometrial serotonin expression levels in the proliferation and secretory phases. Semiquantitative analysis of immunofluorescence was performed using ImageJ (n=12, scale bar=50 μm (bottom) and 100 μm (top), *P<0.05, statistically significant by Student’s *t*-test). (**F**) UMAP plots of hESCs color-coded according to days of decidualization (D0-8). D0 indicates undifferentiated hESCs; D2–8 indicates 2–8 days after 8Br-cAMP and MPA stimulation; WD indicates 48 h after withdrawal of hormones on D8. (**G**) Violin plot showing normalized expression levels of MAOA and MOAB in hESCs after 8Br-cAMP and MPA stimulation. (**H** and **I**) Real-time PCR analysis of the MAOA and MAOB transcriptional expression levels in hESCs after stimulation with 8-Br-cAMP and MPA. The hESCs were collected after 0, 4, 8, 12, 24, 48, and 72 h of stimulation with 8Br-cAMP and MPA, 0 h indicates unstimulated hESCs (n=3, *P<0.05, **P<0.01, ***P<0.001, statistically significant by Student’s *t*-test). (**J**) The protein expression levels of MAOA and MAOB in hESCs after stimulation with 8-Br-cAMP and MPA. The hESCs were collected after 0, 4, 8, 12, 24, 48, and 72 h of stimulation with 8Br-cAMP and MPA, 0 h indicates unstimulated hESCs. MAOA, monoamine oxidase A; MAOB, monoamine oxidase B; Endo, endometrium; Str, stroma; UMAP, uniform manifold approximation and projection; hESCs, human endometrial stromal cells; WD, withdrawal of hormones; 8Br-cAMP, 8-bromoadenosine 3′,5′-cyclic monophosphate; MPA, medroxyprogesterone acetate.

Through spatial transcriptome analysis, MAOA and MAOB levels were found to be significantly increased in the endometrium of the secretory phase compared to the proliferative phase (Fig. 4A). Both MAOA and MAOB expression levels in the endometrial stromal cells were elevated in the secretory phase compared to the proliferative phase, as shown by immunohistochemistry (n = 12) (Fig. 4B and C). Serotonin levels were found to be reduced in the secretory phase compared to the proliferative phase, as shown by immunofluorescence (n = 12) (Fig. 4D and E). We further investigated the changes in the expression levels of MAOA and MAOB at different time points during the *in vitro* induction of decidualization in human endometrial stromal cells. Analysis of single-cell sequencing data revealed that the expression of MAOA and MAOB was significantly increased after the induction of decidualization (Fig. 4F and G). Quantitative real-time PCR (qRT-PCR) also showed that the transcript levels of MAOA and MAOB in stromal cells gradually increased after stimulation with 8Br-cAMP and MPA (n = 3) (Fig. 4H and I). Western blot results showed that the expression levels of MAOA and MAOB in stromal cells increased with time; specifically, the expression levels increased significantly at 48 and 72 h after stimulation (n = 3) (Fig. 4J). This result suggests that MAO may contribute to the decidualization of the endometrium.

### MAO deficiency impairs decidualization in hESCs

**Figure 5.**
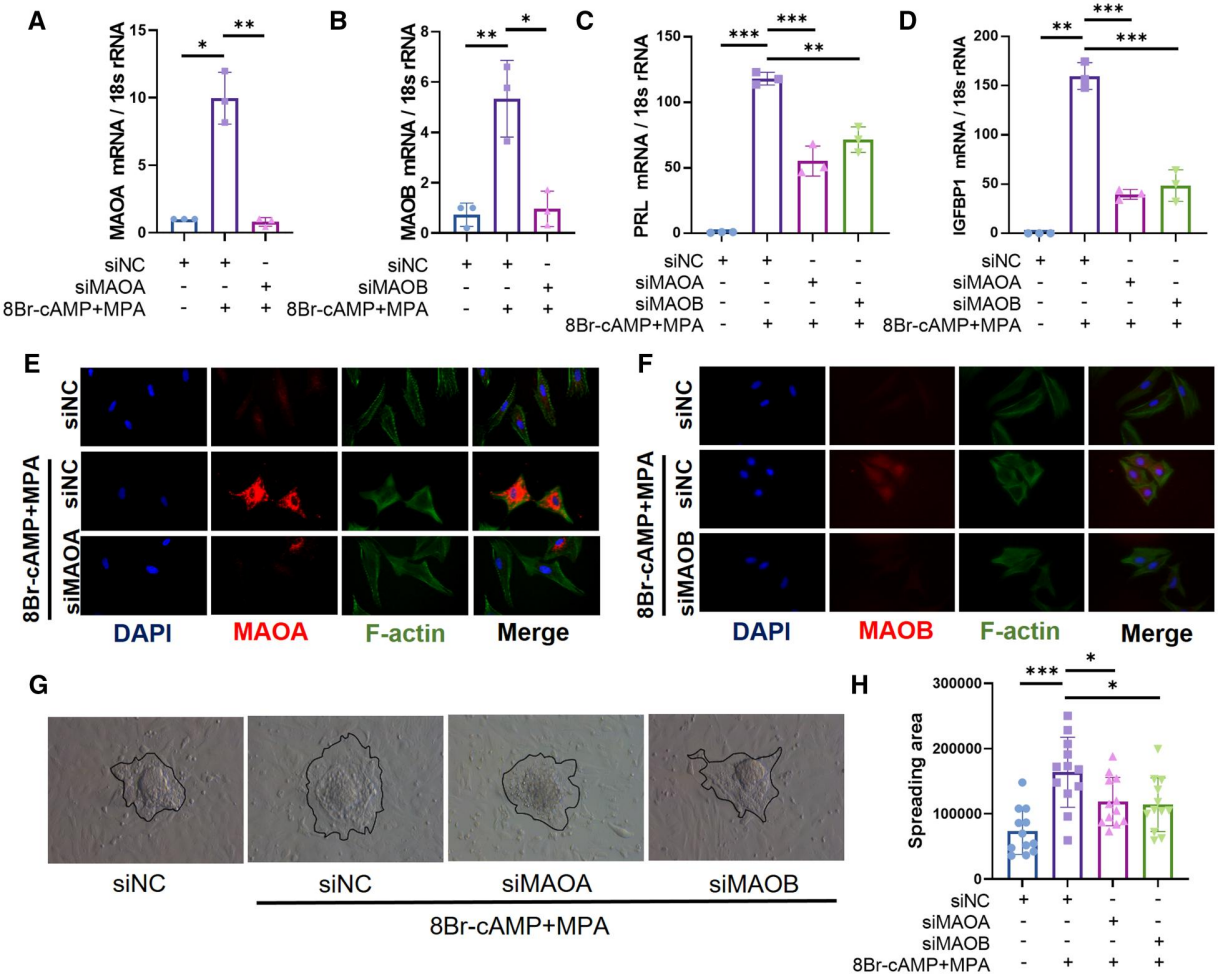
**Knockdown of monoamine oxidase expression impairs *in vitro* human endometrial stromal cells decidualization and trophoblast invasion**. (**A**–**D**) Real-time PCR analysis of the MAOA, MAOB, PRL, and IGFBP1 transcriptional expression levels in hESCs after transfection with siNC, siMAOA, or siMAOB followed by stimulation with 8-Br-cAMP and MPA for 3 days (n=3, *P<0.05, **P<0.01, ***P<0.001, statistically significant by Student’s *t*-test). (**E** and **F**) Immunofluorescence analysis of F-actin, MAOA, and MAOB in hESCs after transfection with siNC, siMAOA, or siMAOB followed by stimulation with 8-Br-cAMP and MPA for 3 days. (**G**) Differentiated endometrial stromal cells were stimulated with 8Br-cAMP+MPA for 3 days and then cocultured with mouse blastocysts, and the invasion of trophoblast cells was observed 2 days later. (**H**) Statistics of the invasion area of trophoblast cells of different groups. The invasion area was quantified using Image-Pro Plus (n=12, *P<0.05, ***P<0.001, statistically significant by ordinary one-way ANOVA). MAOA, monoamine oxidase A; MAOB, monoamine oxidase B; PRL, prolactin; IGFBP1, insulin-like growth factor binding protein 1; hESCs, human endometrial stromal cells; 8Br-cAMP, 8-bromoadenosine 3′,5′-cyclic monophosphate; MPA, medroxyprogesterone acetate.

To investigate the role of MAO in endometrial decidualization, we knocked down MAOA or MAOB by siRNA in human primary endometrial stromal cells before stimulation with 8Br-cAMP and MPA (n = 3) (Fig. 5A and B). After knockdown of MAOA or MAOB, the expression of the decidualization markers prolactin (PRL) and insulin-like growth factor binding protein 1 (IGFBP1) was significantly decreased in decidualized hESCs (Fig. 5C and D). As shown in Fig. 5E and F, decidualized stromal cells displayed polygonal cell morphology with a random distribution of F-actin filaments (n = 3). When endogenous MAOA or MAOB was knocked down, stromal cells maintained a fibroblast-like phenotype upon decidual stimulation. The decidualization of stromal cells facilitates embryonic invasion. Therefore, mouse blastocysts were co-cultured with decidualized endometrial stromal cells for 48 h, after which their invasion capacity was evaluated. The invasion of trophoblasts was inhibited after knockdown of MAO expression in hESCs (n = 12) (Fig. 5G and H), suggesting that MAO participates in regulating the functions of decidualized hESCs.

### MAO deficiency impairs mouse endometrial decidualization

**Figure 6.**
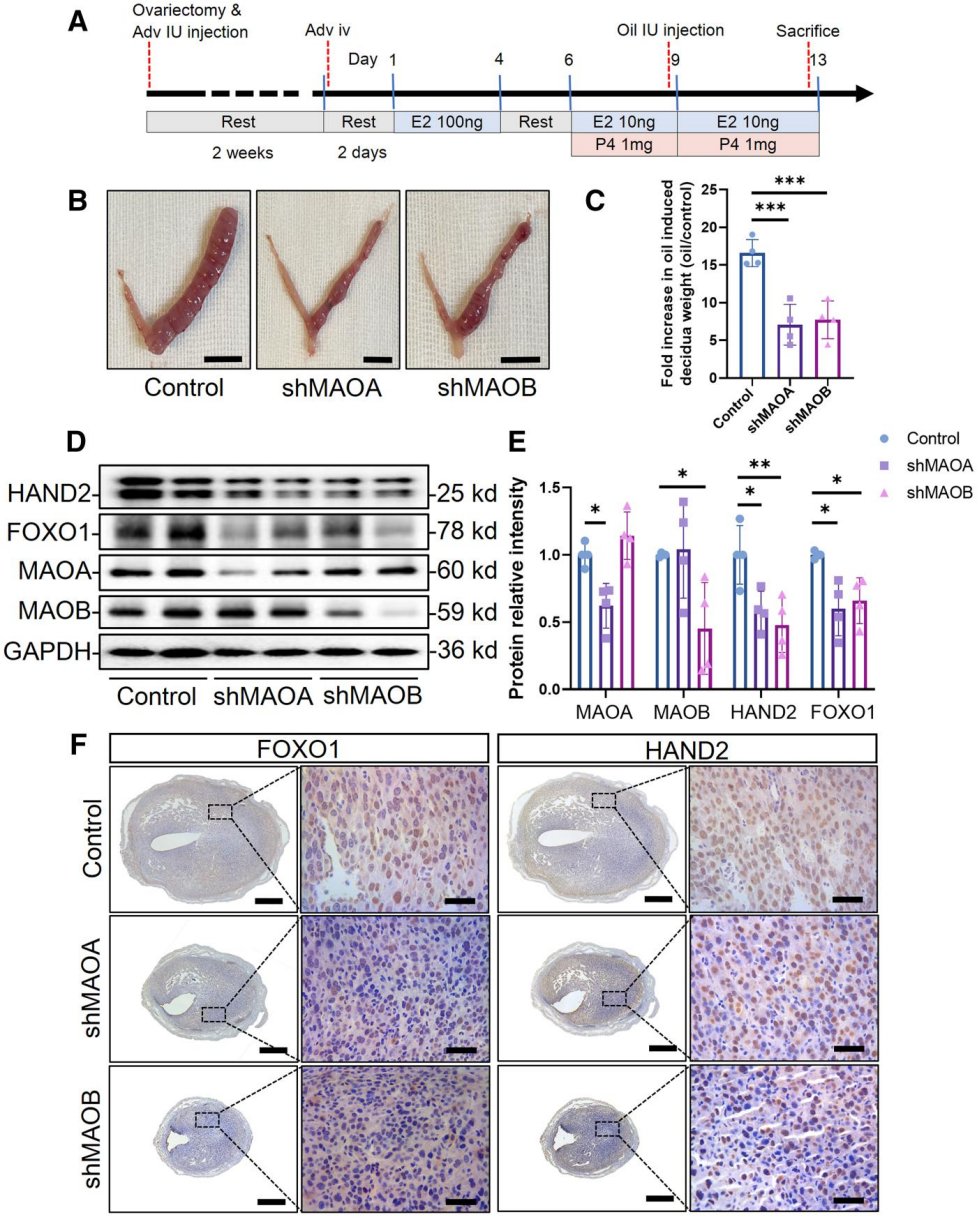
**Knockdown of monoamine oxidase impairs artificially induced endometrial decidualization in mice**. (**A**) Schematic diagram of artificial induction of uterine decidualization in mice. Six-week-old female mice were ovariectomized, and the uterine horns were injected bilaterally with 20 μl of adenovirus, followed by a 2-week rest period. Two weeks later, the mice were injected with adenovirus via the tail vein and rested for two days. Afterwards 50 µl of sesame oil containing 100 ng oestradiol was subcutaneously injected for three consecutive days, followed by 2 days of rest. Then, 50 µl of sesame oil containing 1 mg of progesterone and 10 ng of oestradiol was subcutaneously injected for three consecutive days. Six hours after the third combined administration of estrogen and progesterone, 20 μl of sesame oil was injected into one of the uterine horns (unilaterally) to simulate the decidualization of the endometrium. Subcutaneous injections of estrogen and progesterone were continued for 4 days. Mice were sacrificed 6 h after the last hormone injection. (**B**) The decidualized mouse uteri after knockdown of MAOA or MAOB (bar=1 cm). (**C**) The fold increase in oil-induced decidua weight (weight of the uterus on the oil-induced side/non-induced side) (n=4, ***P<0.001, statistically significant by ordinary one-way ANOVA). (**D**) The protein expression levels of MAOA, MAOB, HAND2, and FOXO1 in uteri after knockdown of MAOA or MAOB. (**E**) Total MAOA, MAOB, HAND2, and FOXO1 protein levels were normalized to total GAPDH levels according to western blot analysis (n=4, *P<0.05, **P<0.01, statistically significant by ordinary one-way ANOVA). (**F**) Immunohistochemistry illustrates FOXO1 and HAND2 expression levels and localization in the uteri after knockdown of MAOA or MAOB (scale bars=500 μm (left) and 50 μm (right)). Adv IU injection, intrauterine injection of adenovirus; Adv iv, tail vein injection of adenovirus; Oil IU injection, intrauterine injection of sesame oil; E2, oestradiol; P4, progesterone; MAOA, monoamine oxidase A; MAOB, monoamine oxidase B; HAND2, heart and neural crest derivatives expressed 2; FOXO1, forkhead box O1.

To further confirm the functions of MAOA and MAOB in endometrial decidualization, we utilized shMAOA and shMAOB adenoviruses to knock down the expression of MAO and artificially induced mouse endometrial decidualization as shown in Fig. 6A. Endometrial decidualization was inhibited in the MAOA and MAOB knockdown groups (Fig. 6B), and the weight ratio of the stimulated side to the non-stimulated side was decreased (Fig. 6C) (n = 4). When endogenous MAOA or MAOB was knocked down, the expression of the decidualization markers FOXO1 and HAND2 decreased (n = 4) (Fig. 6D and E). Immunohistochemistry further confirmed that the uterine expression levels of FOXO1 and HAND2 in the mice were decreased after knockdown of MAOA or MAOB (n = 4) (Fig. 6F). These results suggest that a reduction of endogenous MAOA or MAOB levels in the mouse uterus impairs endometrial decidualization.

### RNA-seq analysis revealed abnormal lipid metabolism in hESCs after MAO knockdown

**Figure 7.**
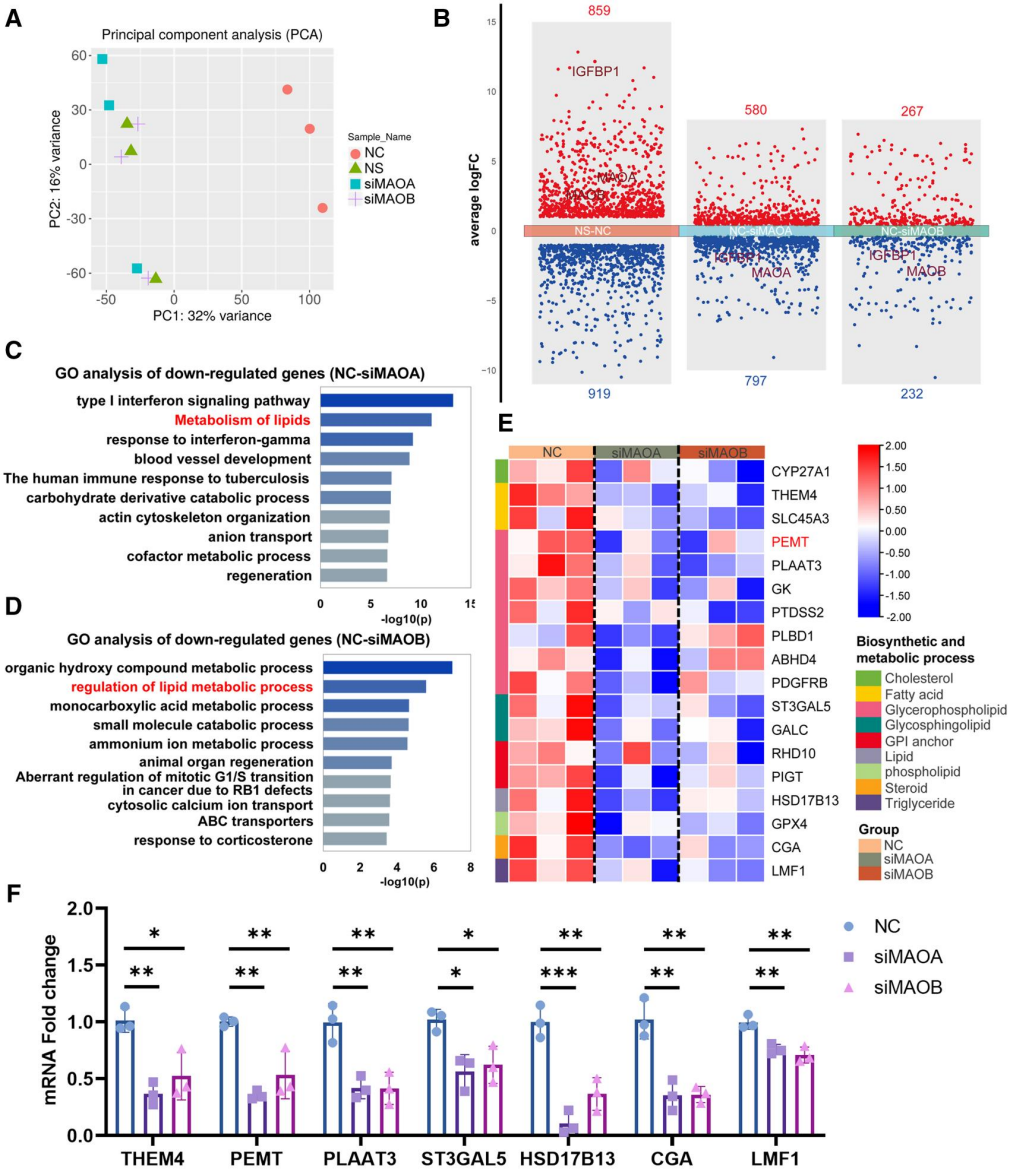
**Knockdown of monoamine oxidase downregulates genes related to lipid metabolism**. (**A**) Principal component analysis of the RNA-seq data from hESCs after transfection with siNC, siMAOA, or siMAOB followed by stimulation with 8-Br-cAMP and MPA for 3 days (corresponding to NC, siMAOA, and siMAOB groups respectively). NS, transfected siNC without 8Br-cAMP+MPA stimulation (n=3). (**B**) Number of DEGs between the different comparisons. (**C** and **D**) GO enrichment analysis of downregulated genes in decidualized hESCs after knockdown of MAOA or MAOB. (**E**) Clustered heatmap of average expression of representative downregulated genes related to lipid metabolism in decidualized hESCs after knockdown of MAOA or MAOB. (**F**) Verification of the expression of genes related to lipid metabolism by real-time PCR in decidualized hESCs after knockdown of MAOA or MAOB, normalized to the 18s rRNA (n=3, *P<0.05, **P<0.01, ***P<0.001, statistically significant by ordinary one-way ANOVA). PCA, principal component analysis; hESCs, human endometrial stromal cells; MAOA, monoamine oxidase A; MAOB, monoamine oxidase B; 8Br-cAMP, 8-bromoadenosine 3′,5′-cyclic monophosphate; MPA, medroxyprogesterone acetate; DEGs, differentially expressed genes; GO, gene ontology.

RNA-seq was performed to further explore the mechanism of impaired decidualization after MAO knockdown in hESCs. Principal component analysis (PCA) showed that there were significant differences between the unstimulated group (NS group) and the 8Br-cAMP+MPA stimulation group (NC group), while the groups with MAOA or MAOB knockdown (siMAOA group and siMAOB group) were also significantly different from the normal decidualization group (n = 3) (Fig. 7A). Differentially expressed gene (DEG) analysis showed that there were series of DEGs in the siMAOA group and siMAOB group compared with the NC group (Fig. 7B). GO analysis showed that the lipid metabolic pathway changed significantly after knockdown of MAOA or MAOB, suggesting that MAO may affect decidualization by regulating lipid metabolism (Fig. 7C and D). A heatmap was constructed to illustrate the downregulation of genes related to lipid metabolism, including genes involved in the synthesis and metabolism of cholesterol, fatty acids, glycerophospholipids, and glycosphingolipids, after knockdown of MAOA or MAOB in hESCs (Fig. 7E). To confirm the accuracy of the RNA-seq results, we selected seven genes for further validation by real-time PCR (n = 3) (Fig. 7F). The validation results were consistent with the RNA-seq results, confirming that lipid metabolism is altered after knockdown of MAOA or MAOB.

### MAO attenuation alters glycerolipid metabolism during decidualization

**Figure 8.**
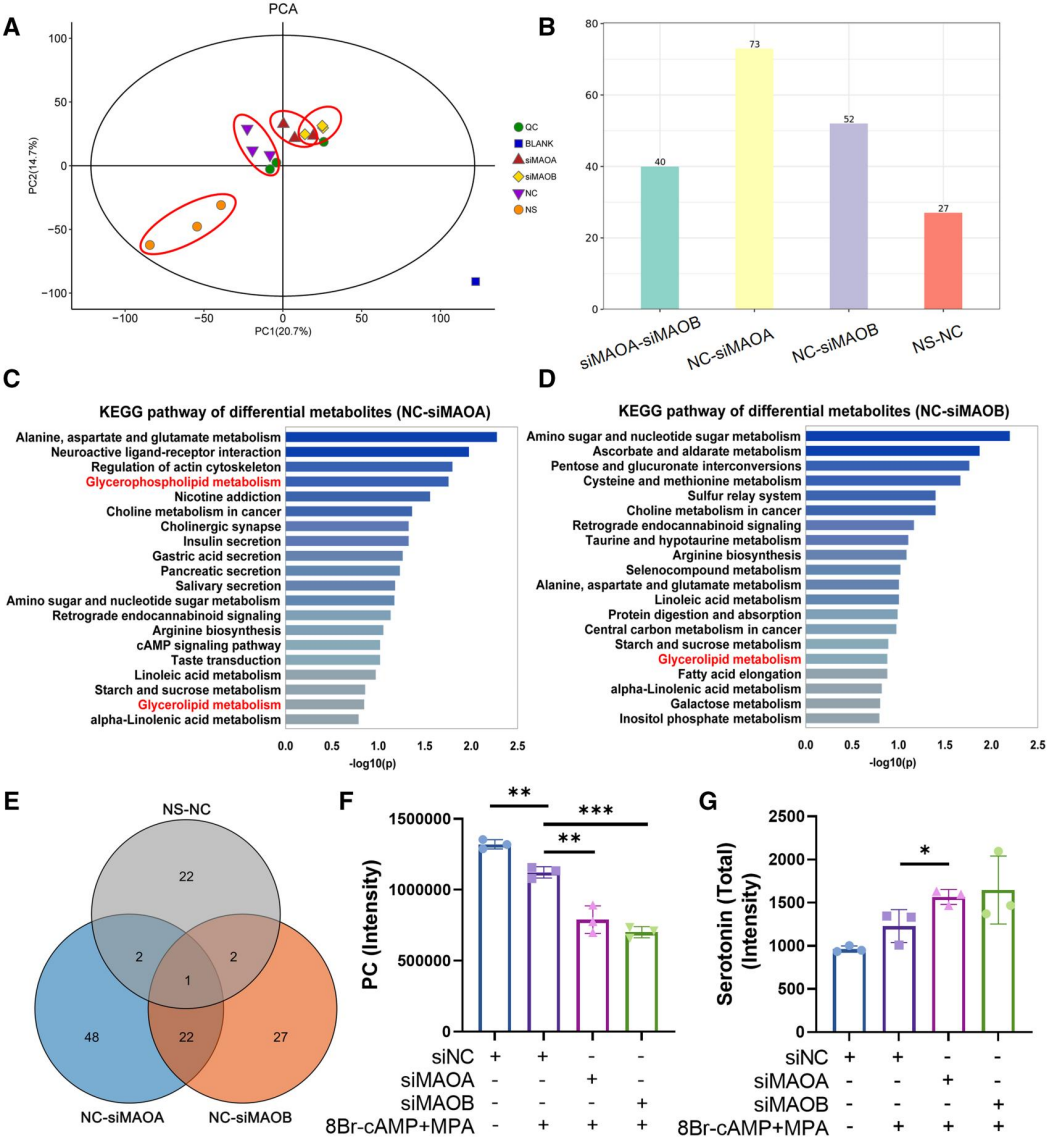
**Altered glycerolipid metabolism in cell supernatants after monoamine oxidase knockdown**. (**A**) PCA analysis of untargeted metabolomics LC/MS analysis data of the cell supernatants of hESCs after transfection with siNC, siMAOA, or siMAOB followed by stimulation with 8-Br-cAMP and MPA for 3 days (corresponding to NC, siMAOA, and siMAOB groups respectively). NS, transfected siNC without 8Br-cAMP+MPA stimulation (n=3). (**B**) Amounts of differentially abundant metabolites in cell supernatants between different comparisons. (**C** and **D**) KEGG enrichment analysis of differentially abundant metabolites in cell supernatants of hESCs after knockdown of MAOA or MAOB. (**E**) Venn diagram showing the overlap in metabolites altered between different comparisons. (**F** and **G**) The expression levels of PC and total serotonin in cell supernatants among different groups detected by untargeted metabolomics LC/MS analysis (n=3, *P<0.05, **P<0.01, ***P<0.001, statistically significant by Student’s *t*-test). PCA, principal component analysis; LC/MS, liquid chromatography-mass spectrometry; hESCs, human endometrial stromal cells; MAOA, monoamine oxidase A; MAOB, monoamine oxidase B; 8Br-cAMP, 8-bromoadenosine 3′,5′-cyclic monophosphate; MPA, medroxyprogesterone acetate; KEGG, Kyoto Encyclopedia of Genes and Genomes; PC, phosphatidylcholine.

Since RNA-seq showed that MAO mediates lipid metabolism, we collected the corresponding cell culture supernatants during decidualization for metabolite detection. PCA showed that the supernatant of cells stimulated without 8Br-cAMP+MPA was significantly different from that of the other groups, and there were also series of differentially abundant metabolites between the NC group and the siMAOA and siMAOB groups (n = 3) (Fig. 8A and B). Kyoto Encyclopedia of Genes and Genomes (KEGG) enrichment analysis of the differentially expressed metabolites showed that the glycerophospholipid or glycerolipid metabolic pathways were regulated by MAO (Fig. 8C and D). We compared the differentially abundant metabolites between different groups by Venn analysis and found that PC differed significantly among the three group during the decidualization induction process and knockdown of MAO (Fig. 8E). As illustrated in Fig. 8F, decidualization-impaired hESCs after MAOA or MAOB knockout exhibited abnormal phospholipid metabolic pathways, with an especially significant decrease in PC in the cell supernatant (Fig. 8F), suggesting a potential role for PC in endometrial decidualization.

### PC supplementation rescues serotonin-suppressed decidualization

**Figure 9.**
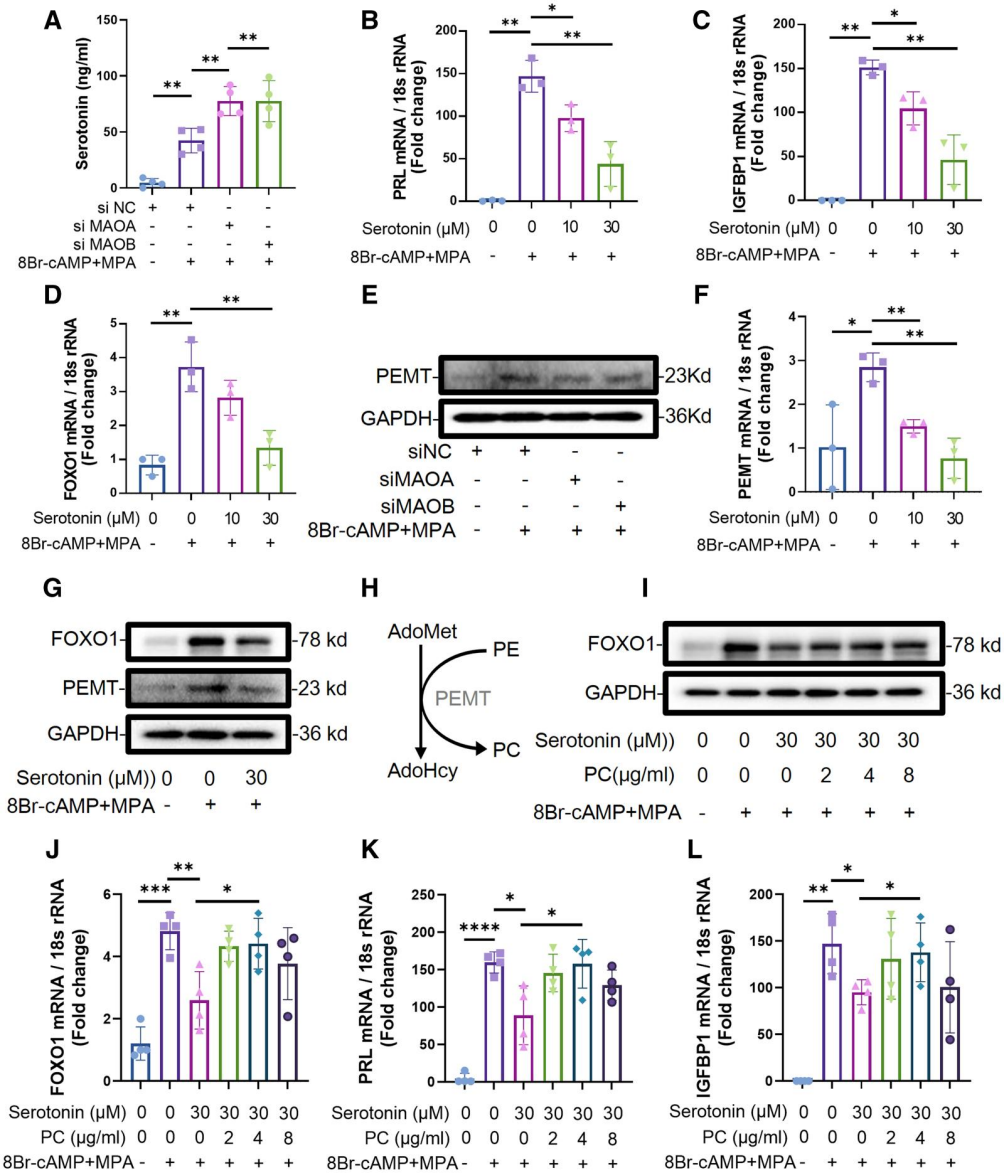
**Phosphatidylcholine rescues serotonin-suppressed human endometrial stromal cell decidualization**. (**A**) ELISA was used to detect the expression of serotonin in the cell supernatant of hESCs after knockdown of MAOA or MAOB followed by stimulation with 8-Br-cAMP and MPA for 3 days (n=4, **P<0.01). (**B**–**D**) Real-time PCR analysis of the PRL, IGFBP1, and FOXO1 transcriptional expression levels in hESCs treated with different doses of serotonin followed by stimulation with 8-Br-cAMP and MPA for 3 days (n=3, *P<0.05, **P<0.01, statistically significant by Student’s *t*-test). (**E**) The protein expression levels of PEMT in hESCs after transfection with siNC, siMAOA, or siMAOB followed by stimulation with 8-Br-cAMP and MPA for 3 days. (**F**) Real-time PCR analysis of the PEMT transcriptional expression levels in hESCs treated with serotonin followed by stimulation with 8-Br-cAMP and MPA for 3 days (n=3, *P<0.05, **P<0.01, statistically significant by Student’s *t*-test). (**G**) The protein expression levels of PEMT and FOXO1 in hESCs treated with serotonin followed by stimulation with 8-Br-cAMP and MPA for 3 days. (**H**) Synthesis of phosphatidylcholine from phosphatidylethanolamine catalyzed by PEMT. (**I**) The protein expression levels of FOXO1 in hESCs treated with 30 μM serotonin and different doses of PC followed by stimulation with 8-Br-cAMP and MPA for 3 days. (**J**–**L**) Real-time PCR analysis of the PRL, IGFBP1, and FOXO1 transcriptional expression levels in hESCs treated with 30 μM serotonin and different doses of PC followed by stimulation with 8-Br-cAMP and MPA for 3 days (n=4, *P<0.05, **P<0.01, ***P<0.001, ****P<0.0001, statistically significant by Student’s *t*-test). hESCs, human endometrial stromal cells; MAOA, monoamine oxidase A; MAOB, monoamine oxidase B; PRL, prolactin; IGFBP1, insulin-like growth factor binding protein 1; FOXO1, forkhead box O1; 8Br-cAMP, 8-Bromoadenosine 3′,5′-cyclic monophosphate; MPA, medroxyprogesterone acetate; PEMT, phosphatidylethanolamine *N*-methyltransferase; PC, phosphatidylcholine; PE, phosphatidylethanolamine.

To investigate how MAO deficiency leads to the reduction of PC, we found that PEMT, which is a kind of PC synthase, was downregulated in DEGs by RNA-seq (Fig. 7E). However, the mechanism by which MAO regulates the expression of lipid metabolism-related genes remains unclear. An analysis of the metabolic substrates of MAO through LC/MS metabolome analysis indicated that the expression level of serotonin increased after knockdown of MAO, which was further confirmed by ELISA (n = 4) (Figs 8G and 9A). Previous studies have suggested that serotonin impairs decidualization in rats. The qPCR results also confirmed that serotonin stimulation in hESCs resulted in a decrease in the decidualization marker molecules PRL and IGFBP1 (n = 3) (Fig. 9B and C). FOXO1 is vital for the induction of decidual markers including PRL and IGFBP1 in hESCs. As shown in Fig. 9D and G, serotonin administration led to the downregulation of FOXO1 (n = 3). Moreover, the expression of PEMT decreased after stimulation with serotonin (n = 3) (Fig. 9F and G), which was consistent with the knockdown of MAOA or MAOB (Fig. 9E). Since PEMT catalyzes the synthesis of phosphatidylethanolamine (PE) to PC, this finding explains the decreased levels of PC after knockdown of MAOA or MAOB (Fig. 9H). To further confirm the role of PC in decidualization, we added exogenous PC to investigate whether serotonin-suppressed decidualization could be improved. The qRT-PCR and WB results showed that the expression of PRL, IGFBP1, and FOXO1 increased after supplementation with PC (n = 4) (Fig. 9I–L).

### Overexpression of FOXO1 partially rescues decidualization defects caused by knockdown of MAO or treatment with serotonin

**Figure 10.**
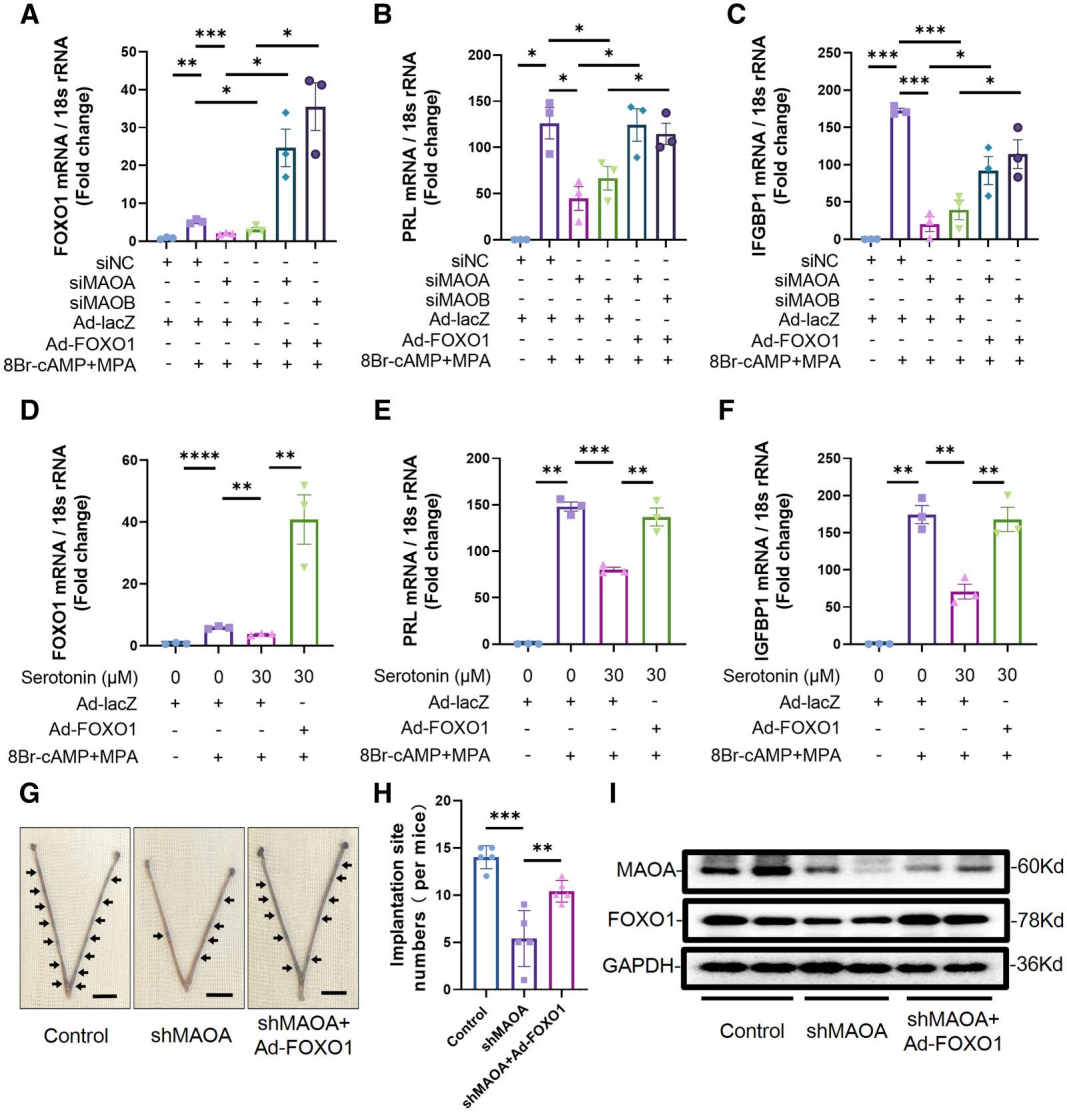
**Overexpression of forkhead box O1 partially rescues the impaired decidualization caused by knockdown of monoamine oxidase or treatment with serotonin**. (**A**–**C**) Real-time PCR analysis of the PRL, IGFBP1, and FOXO1 transcriptional expression levels in hESCs after transfection with siNC, siMAOA, or siMAOB and infection with Ad-FOXO1 or Ad-lacZ for 2 days followed by stimulation with 8-Br-cAMP and MPA for 3 days (n=3, *P<0.05, **P<0.01, ***P<0.001, statistically significant by Student’s *t*-test). (**D**–**F**) Real-time PCR analysis of the PRL, IGFBP1, and FOXO1 transcriptional expression levels in hESCs after infection with Ad-FOXO1 or Ad-lacZ for 2 days followed by treatment with 30 μM serotonin and stimulation with 8-Br-cAMP and MPA for 3 days (n=3, **P<0.01, ***P<0.001, ****P<0.0001, statistically significant by Student’s *t*-test). (**G**) Adenovirus was injected into the bilateral uterine horns of mice at dpc1.5 to knockdown MAOA or overexpress FOXO1. Chicago blue dye was injected into the tail vein at dpc4.5 to observe the embryo implantation sites. Representative images of embryo implantation sites in the uteri at dpc4.5 from the control group, the shMAOA group, and the shMAOA+Ad-FOXO1 group. Arrow shows embryo implantation site visualized by Chicago Blue dye (bar=1 cm). (**H**) The average number of embryo implantation sites among different groups (n=5, **P<0.01, ***P<0.001, statistically significant by Student’s *t*-test). (**I**) The protein expression levels of MAOA and FOXO1 in uteri at dpc4.5 from the control group, the shMAOA group, and the shMAOA+Ad-FOXO1 group. PRL, prolactin; IGFBP1, insulin-like growth factor binding protein 1; FOXO1, forkhead box O1; hESCs, human endometrial stromal cells; MAOA, monoamine oxidase A; MAOB, monoamine oxidase B; 8Br-cAMP, 8-bromoadenosine 3′,5′-cyclic monophosphate; MPA, medroxyprogesterone acetate; Ad-FOXO1, adenovirus overexpressing FOXO1; Ad-lacZ, control adenovirus; dpc1.5/dpc4.5, 1.5/4.5 days post-coitum.

Since serotonin inhibited the expression of FOXO1 in hESCs, we further explored whether the overexpression of FOXO1, a key molecule in decidualization, could rescue the impaired decidualization. qRT-PCR results showed that FOXO1 overexpression could partially rescue the decreased expression levels of PRL and IGFBP1 caused by knockdown of MAOA or MAOB (n = 3) (Fig. 10A–C). Decidualization of hESCs suppressed by serotonin was also rescued by the overexpression of FOXO1 (n = 3) (Fig. 10D–F).

The results of this study suggest that the decreased expression of MAO in patients with RIF may be an important cause of embryo implantation failure. Therefore, we wondered whether the overexpression of FOXO1 could rescue embryo implantation failure induced by this factor. The number of mouse embryo implantation sites was significantly reduced after MAOA knockdown (n = 5), while the overexpression of FOXO1 partially rescued embryo implantation in mice (Fig. 10G–I). These results show that overexpression of FOXO1 rescues the decidualization defects caused by attenuated MAO or excess serotonin and improves embryo implantation.

## Discussion

**Figure 11.**
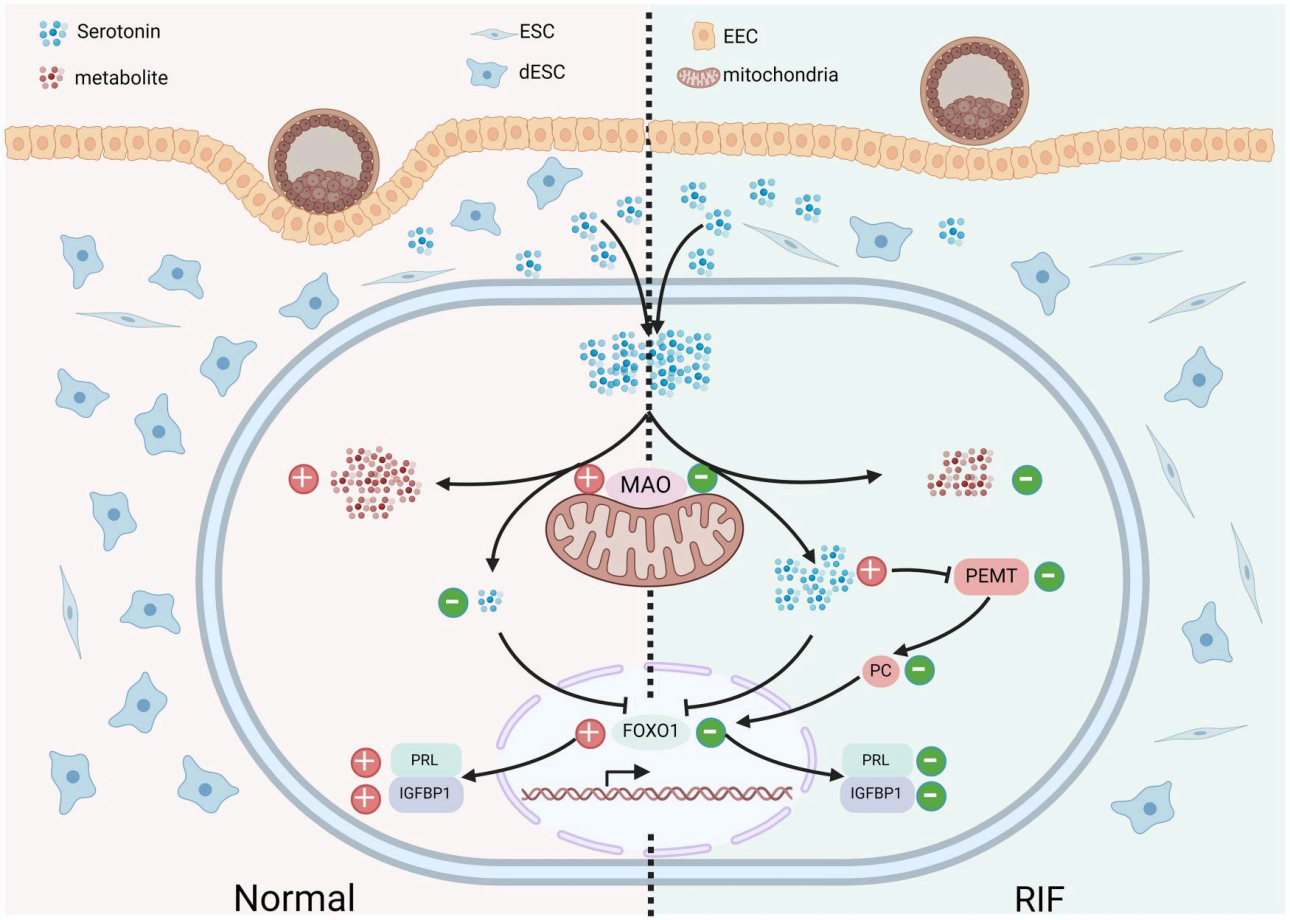
**Schematic representation of the role of monoamine oxidase in regulating decidualization in patients with recurrent implantation failure and fertile controls**. The serotonin metabolic pathway is impaired due to attenuated MAO in the endometrial stromal cells of patients with RIF. Excessive serotonin leads to a decrease in FOXO1 as well as the decidualization markers PRL and IGFBP1. Attenuated MAO or excessive serotonin induces a decrease in PEMT, a gene related to phospholipid metabolism, and a corresponding decrease in PC. Exogenous PC upregulates FOXO1 and induces an increase in PRL and IGFBP1. RIF, recurrent implantation failure; ESC, non-decidualized endometrial stromal cells; dESC, decidualized endometrial stromal cells; EEC, endometrial epithelial cells; MAO, monoamine oxidase; PRL, prolactin; IGFBP1, insulin-like growth factor binding protein 1; FOXO1, forkhead box O1; PEMT, phosphatidylethanolamine *N*-methyltransferase; PC, phosphatidylcholine.

Patients undergoing ART are more likely to experience stress and anxiety, which are thought to affect embryo implantation (Turner *et al.*, 2013; Cesta *et al.*, 2016). However, the exact mechanism linking psychological stress with embryo implantation failure is unknown, and treatment options are limited. This study found excessive serotonin, a neurotransmitter closely associated with mental health, in the endometrium of patients with RIF. Impaired serotonin metabolism due to attenuated MAO, an enzyme involved in the metabolism of serotonin, contributes to defective decidualization in endometrial stromal cells. Our findings indicate that elevated levels of MAO promote decidualization by regulating phospholipid metabolism during the mid-secretory phase of the menstrual cycle. Our *in vitro* study revealed that decidualization suppressed by either excess serotonin or reduced MAO can be rescued by upregulating FOXO1 through exogenous PC supplementation. Taken together, we propose that defective metabolism of the neurotransmitter serotonin is an important factor leading to RIF, providing a new perspective and potential treatment options for infertility linked to anxiety and depression (Fig. 11).

Multiple studies have shown that the failure of embryo implantation in patients with RIF is mutually causal with the associated anxiety and depression, as they mutually reinforce each other (Wang *et al.*, 2021; Zhao *et al.*, 2022). However, the specific mechanisms are not clear. Anxiety and depression are common mental health problems, and there is a close relationship between them and monoamine neurotransmitters, which can affect endometrium receptivity. Previous studies have shown that abnormalities in adrenergic receptor signaling affect decidualization and embryo implantation in patients with RIF (Wu *et al.*, 2022), and that uterine serotonin injection inhibits decidualization in rats through an unknown mechanism (Mitchell *et al.*, 1983). In this study, we found excessive serotonin in the endometrial stromal cells of patients with RIF. Single-cell transcriptomic analysis demonstrated that the serotonin synthesis-related genes TPH1 and TPH2 were barely expressed during proliferative and secretory phases, while the expression levels of the serotonin metabolism-related genes MAOA and MAOB were significantly increased during the mid-secretory phase. Our data demonstrated that the expression levels of MAO are reduced in the endometrium of patients with RIF, which may lead to impaired serotonin metabolism and, therefore, excess serotonin. In this study, we elucidated the relationship and possible mechanisms between psychological stress and embryo implantation failure in RIF.

Lipid metabolism plays an important role in the process of endometrial decidualization (Ye *et al.*, 2021). The differentiation of stromal cells requires the utilization of glucose and fatty acids (Tsai *et al.*, 2014; Dean, 2019; Tamura *et al.*, 2021). Inhibition of fatty acid β-oxidation can prevent the decidualization of stromal cells (Tsai *et al.*, 2014). On the other hand, molecules such as lipids may function through specific receptors. For example, the binding of lysophosphatidic acid (LPA) to LPA receptor 3 (LPA3) in the endometrium stimulates cyclooxygenase 2 expression, further promoting decidualization by upregulating WNT4 and BMP 2 (Aikawa *et al.*, 2017). A variety of phospholipids (PC, phosphatidylinositol, phosphatidylglycerol, and diglyceride) were found to be elevated in the uterine cavity of pregnant ewes, indicating the importance of phospholipids in early pregnancy (O’Neil and Spencer, 2021). Phosphatidic acid (PA) treatment can promote AKT binding to PP2A in endometrial stromal cells, reducing the phosphorylation of AKT and thereby also reducing the phosphorylation of FOXO1, and promoting decidualization (Lee *et al.*, 2016). By combining RNA-seq and metabolite detection, we found that MAO is involved in the regulation of phospholipid metabolism, like PC, which has been shown to be important for decidualization. It is reported that PC is predominantly altered in endometrial fluid from implanted and non-implanted IVF cycles, suggesting its importance for uterine receptivity (Matorras *et al.*, 2020). In this study, we showed that PC supplementation can rescue the impaired decidualization inhibited by excessive serotonin, providing new evidence of the involvement of phospholipids in endometrial receptivity.

Previous studies have suggested that intralipid can be used as an alternative therapy for patients with RIF (Coulam, 2021), which is accompanied by an altered profile of plasma phospholipids, specifically PC, after treatment (Canella *et al.*, 2023). Through *in vitro* experiments, it was also confirmed that PC supplementation rescues serotonin-suppressed decidualization. Intravenous or intrauterine PC supplementation may become a potential therapy to improve endometrial receptivity in some patients with RIF. However, more safety testing including the effects of PC on mothers and offspring in animal studies is needed to support the potential translational use of PC in RIF treatment. In addition, the underlying mechanism of improvement of endometrial decidualization after PC treatment also needs further exploration.

FOXO1 is a decidualization marker in endometrial stromal cells and genes that are specifically regulated during decidualization are expressed abnormally as a result of FOXO1 reduction. This study also revealed that either excessive serotonin or attenuated MAO leads to impaired decidualization by inhibiting FOXO1 expression. Impaired decidualization of hESCs caused by excessive serotonin or attenuated MAO was successfully rescued by exogenous overexpression of FOXO1, providing a potential target for the treatment of patients with RIF.

The identification of patients with RIF is challenging until they have experienced multiple failed embryo implantations. Studies have shown that endometrial thickness on the day of embryo transfer is a poor predictor of IVF treatment outcome (Griesinger *et al.*, 2018). Therefore, it is of great clinical significance to find new and reliable molecular biomarkers for early diagnosis of RIF for clinical diagnosis and treatment. Through ROC analysis, we found that the expression levels of MAOA, MOAB, and serotonin have potential predictive value for RIF. In particular, the expression levels of MAOA and MAOB in endometrial stromal cells, as detected by immunohistochemistry, showed better predictive value for RIF. Identifying potential patients with RIF through the expression levels of MAOA, MOAB, or serotonin may help in timely adjustment of clinical strategies and improvement of outcomes. Furthermore, our ongoing clinical trials are exploring whether measuring serotonin levels in the blood and endometrium can predict endometrial receptivity in patients with RIF. If successful, serotonin testing may offer a quicker and less invasive approach for predicting the implantation window and endometrial receptivity.

Taken together, these findings offer new insights into the mechanism of serotonin homeostasis in human endometrial decidualization and provide a firm experimental basis for future research on the relationship between psychological stress and the early adverse outcomes in RIF. MAO plays a vital role in decidualization by regulating phospholipid metabolism via serotonin. These findings indicate that dysregulation of serotonin homeostasis and MAO is related to RIF and point towards potentially valuable treatments for these patients.

## Supplementary Material

hoae042_Supplementary_Data

## Data Availability

The data underlying this article will be shared on reasonable request to the corresponding author (Dr Sheng). RNA-seq information and raw data can be found at the NCBI BioProject number PRJNA892255.
