# Supplementary material for: Dysregulation of endometrial stromal serotonin homeostasis leading to abnormal phosphatidylcholine metabolism impairs decidualization in patients with recurrent implantation failure
Source: Hum Reprod Open. 2024 Jun 20;2024(3):hoae042. doi: 10.1093/hropen/hoae042 (PMC11293872; doi:10.1093/hropen/hoae042)
Supplement: hoae042_Supplementary_Data [file hoae042_supplementary_data.docx]

**Supplementary Table S1:** RT–qPCR primers used in the study

| Gene symbol | Forward primer (5′–3′) | Reverse primer (5′–3′) |
| --- | --- | --- |
| *MAOA* | ACCATCCTGTCACTCACGTT | CCATTGGAAGCCGCTGAATT |
| *MAOB* | ATGACATGGGGCGAGAGATT | CACAGGAACCAGAGAGCAGA |
| *PRL* | CACTACATCCATAACCTCTC | ATGCTGACTATCAAGCTCAG |
| *IGFBP1* | TATGATGGCTCGAAGGCTCTC | GTAGACGCACCAGCAGAGTC |
| *FOXO1* | GGATGTGCATTCTATGGTGTACC | TTTCGGGATTGCTTATCTCAGAC |
| *PEMT* | CTGGAATGTGGTTGCACGATG | GCTTAGAGAGTAGCAGGCCA |
| *THEM4* | GAACAAGGACCTAAGACTGCTC | AGAACATCACGTATTCAAAGCCC |
| *PLAAT3* | TTTTCGCCCTTTCTACAGACAC | CCTGCGACCTCACTTGGAG |
| *ST3GAL5* | AGGAATGTCGTCCCAAGTTTG | GGAGTAAGTCCACGCTATACCT |
| *HSD17B13* | CCTACTTGGAGTCGTTGGTGA | CCAATATGCTCTGTCGTTTTGC |
| *CGA* | TGCCCAGAATGCACGCTAC | TTGGACCTTAGTGGAGTGGGA |
| *LMF1* | GGCGGAAGACTGGGTACTC | CACCAGGAATGCCACGAAG |
| *18S rRNA* | CGGCTACCACATCCAAGGAA | CTGGAATTACCGCGGCT |
